# Protective effects and regulatory pathways of melatonin in traumatic brain injury mice model: Transcriptomics and bioinformatics analysis

**DOI:** 10.3389/fnmol.2022.974060

**Published:** 2022-09-09

**Authors:** Jiayuanyuan Fu, Qiang Zhou, Biying Wu, Xuekang Huang, Zhaohua Tang, Weilin Tan, Ziyu Zhu, Mengran Du, Chenrui Wu, Jun Ma, Ehab Balawi, Z. B. Liao

**Affiliations:** Department of Neurosurgery, The First Affiliated Hospital of Chongqing Medical University, Chongqing, China

**Keywords:** traumatic brain injury, melatonin, lncRNA, microRNA, circular RNA

## Abstract

Traumatic brain injury (TBI) is the leading cause of disability and mortality globally. Melatonin (Mel) is a neuroendocrine hormone synthesized from the pineal gland that protects against TBI. Yet, the precise mechanism of action is not fully understood. In this study, we examined the protective effect and regulatory pathways of melatonin in the TBI mice model using transcriptomics and bioinformatics analysis. The expression profiles of mRNA, long non-coding RNA (lncRNA), microRNA (miRNA), and circular RNA (circRNA) were constructed using the whole transcriptomes sequencing technique. In total, 93 differentially expressed (DE) mRNAs (DEmRNAs), 48 lncRNAs (DElncRNAs), 59 miRNAs (DEmiRNAs), and 59 circRNAs (DEcircRNAs) were identified by the TBI mice with Mel treatment compared to the group without drug intervention. The randomly selected coding RNAs and non-coding RNAs (ncRNAs) were identified by quantitative real-time polymerase chain reaction (qRT-PCR). To further detect the biological functions and potential pathways of those differentially expressed RNAs, Kyoto Encyclopedia of Genes and Genomes (KEGG) and Gene Ontology (GO) analyses were executed. In our research, the regulatory network was constructed to show the relationship of lncRNA-RBPs. The lncRNA-mRNA co-expression network was established based on the Pearson coefficient to indicate the expression correlations. Moreover, the DEcircRNA–DEmiRNA–DEmRNA and DElncRNA–DEmiRNA–DEmRNA regulatory networks were constructed to demonstrate the regulatory relationship between ncRNAs and mRNA. Finally, to further verify our predicted results, cytoHubba was used to find the hub gene in the synaptic vesicle cycle pathway, and the expression level of SNAP-25 and VAMP-2 after melatonin treatment were detected by Western blotting and immunofluorescence. To sum up, these data offer a new insight regarding the molecular effect of melatonin treatment after TBI and suggest that the high-throughput sequencing and analysis of transcriptomes are useful for studying the drug mechanisms in treatment after TBI.

## Introduction

Traumatic brain injury (TBI) is the leading cause of mortality and disability in all trauma-related injuries worldwide (Dewan et al., 2018). In the United States, there is an estimated rate of 1.7 million occurrences of TBI every year (Pundlik et al., 2020). TBI is the most common cause of mortality and disability in the population aged <40 years old (Maas et al., 2017). In addition, rates of death and morbidity caused by TBI in undeveloped countries have also been increasing (Khellaf et al., 2019).

A variety of treatments have been developed for TBI. The selection of treatment depends on the severity injury. For example, neuroprotective therapies, which can improve behavioral outcomes and limit secondary tissue loss, have been well established in multiple animal models of TBI (Khellaf et al., 2019). Yet, the translation of neuroprotective strategies to the clinical setting has been disappointing.

Melatonin (Mel), N-Acetyl-5-methoxytryptamine, a hormone synthesized and secreted by the pineal gland, acts both centrally and peripherally (Ikram et al., 2021). Previous studies have reported that Mel can be used to treat TBI (Osier et al., 2018). Studies have indicated that Mel can act against inflammation (Dehghan et al., 2018), alleviate oxidative damage (Salman et al., 2021), and inhibit neuronal apoptosis (Wu et al., 2016) in TBI. Moreover, Wu et al. (2022) in our group indicated the potential mechanism of Mel on anti-ferroptosis in TBI. Yet, the concrete protective mechanism of Mel in TBI is still not fully understood.

The non-coding portion of the genome, including microRNAs (miRNAs), long non-coding RNAs (lncRNA), circular RNAs (circRNA), and extracellular RNAs (Matsui and Corey, 2017), is crucial for normal development and disease. These RNAs control different gene expression levels, involving chromatin architecture, transcription, RNA splicing, editing, and translation, and are involved in a variety of biological functions (Mattick and Makunin, 2006). For example, Di Pietro et al. (2017) suggested that several miRNAs could be used as valid biomarkers for the diagnosis of severe TBI according to TaqMan sequencing in the serum of TBI patients. In addition, Wang et al. (2017) discovered 271 differently expressed lncRNAs post-TBI, these lncRNAs have a role in inflammation, DNA transcription, and apoptosis. Jiang et al. (2019) profiled 191 differentially expressed circRNAs in the CCI model, which may be related to inflammation, cell death, and repair of damage. The previous study of our group revealed the protective role of circLphn3 in the blood-brain barrier after TBI (Cheng et al., 2022) and identified the circPtpn14/miR-351-5p/5-LOX axis might be a new mechanism regulating the effect of Mel against TBI (Wu et al., 2022), in addition, we found that to inhibit circIgfbp2 could alleviate the synapse dysfunction caused by mitochondrial dysfunction and oxidative stress through the miR-370-3p/BACH1/HO-1 axis after TBI (Du et al., 2022). Nevertheless, there is a lack of studies investigating the transcriptomics analysis of Mel intervention in TBI.

In this study, transcriptomics sequencing was used to profile RNAs alternations that occurred in melatonin treatment of TBI. Furthermore, we performed Kyoto Encyclopedia of Genes and Genomes (KEGG) and Gene Ontology (GO) analyses to identify the potential signaling pathways and the biological function of these RNAs with differential expression. And the regulatory networks were constructed to show the interaction between the differentially expressed RNAs. Finally, to further verify our KEGG results, the hub gene in the significant pathway was identified through Western blotting and immunofluorescence. The present study provides broader and more novel insight into the mechanism of the drug at the molecular level of TBI treatment with melatonin.

## Materials and methods

### Animals

C57BL/6 male mice (6–8 weeks) were purchased from the Chongqing Medical University Animal Experiment Center (Chongqing, China). All animal studies (including the mice euthanasia procedure) were done in compliance with Chongqing Medical University animal care regulations and conducted according to the American Association for Accreditation of Laboratory Animal Care and the Institutional Animal Care and Use Committee guidelines (No. 2021-177). All mice were housed in an individually ventilated cages (IVC) environment, setting the temperature to 22 ± 1°C, relative humidity to 50 ± 1%, a light/dark cycle to 12/12 h, and they were given water and food *ad libitum*.

The animal sample size was determined using a sample size calculator.^1^ Mice were randomly divided into 3 groups: sham group, TBI group, and TBI + Mel group. A total of 45 mice (15 mice/group) were performed the modified neurological severity score test and evaluated the neural function at 0, 1, and 3 days, 9 mice (3 mice/group) were performed for RNA sequencing at 3 days. The rest of the mice (5 mice/group) were sacrificed to obtain the brain tissue for the following Western blotting and immunofluorescence. A 5% isoflurane was used to induce initial sleep and 1.5% isoflurane for sustaining anesthesia in oxygen-enriched air (20% oxygen/80% air) with spontaneous ventilation.

Traumatic brain injury mouse model was constructed by performing controlled cortical impact (CCI), and an electronically controlled pneumatic impact device (PSI, United States) was employed to establish the mice CCI injury model. To be brief, mice were placed on the stereotactic frame. The head was assembled on the foreside, and equipped with a built-in heating bed to maintain the mice’s body temperature at 37°C. The next step is to make a longitudinal incision on the midline of the mice skull under the aseptic condition, a portable trephine was employed over the left parietal cortex for craniotomy (the coordinate center of the craniotomy relative to bregma, the position is: posterior 2 mm, lateral 2 mm), after which to remove the bone flap. The pneumatic cylinder with a flat tip (3 mm in diameter) was used to generate the CCI model (velocity: 5.0 m/s, depth: 1.5 mm, and impact duration: 100 ms). To close the scalp using cyanoacrylate tissue glue. The sham mice group was treated with scalp incisions without removing the bone flap. According to the grasping test of the contralateral limb after 2 h of TBI, we chose the successful TBI model mice for the next experiment. Melatonin was administered intraperitoneally (i.p.) after TBI as a single dose of 10 mg/kg once daily for 3 days.

### Neurobehavioral evaluation

The modified neurological severity score test (mNSS test) (Chen et al., 2001), which included motor (2 points), sensory (3 points), balance (6 points), and reflex and abnormal movement (4 points), was used to evaluate the neural function in mice at 1, 3 days after drug injection. The maximum deficit score was 18 points, with a score of 1∼6 points representing mild damage, 7∼12 points representing moderate damage, and 13∼18 points representing severe damage. The sheet is shown in Supplementary Table 1

### RNA library construction and sequencing

Three days after the surgery, brain samples (ipsilesional cortex) were collected and stored in frozen liquid nitrogen for further RNA sequencing (BGI, Shenzhen, China). BGI was used to perform the library construction and sequencing.

Magnetic beads attached with Oligo (dT) were used to purify mRNA. The purified mRNA of 3 μg was splintered into small pieces at 4°C with fragment buffer. A random hexamer primed reverse transcription was used to produce the first-strand cDNA and generate the second-strand cDNA synthesis. A repair was ended by adding the A-Tailing Mix and RNA Index Adapters *via* incubation. The previous step was amplified by PCR, after which cDNA fragments were obtained, which was followed by purification of the products by Ampure XP Beads, and dissolving in EB solution. The product was validated on the Agilent Technologies 2100 bioanalyzer. The PCR products with double-stranded from the former step were heated and denatured, after which the splint oligo sequence was used to circularize to obtain the final library. The final library was from formatting the single-strand circle DNA (ssCir DNA). The library was amplified by phi29 to get DNA nanoball (DNB) with over 300 copies of one molecule. The DNBs were loaded into the patterned nanoarray, and a pair was produced on the BGIseq-500 platform (BGI).

Approximately, 1 μg total RNA per sample was treated with Ribo-Zero™ Magnetic Kit (Epicentre) to deplete rRNA. The retrieved RNA was fragmented by adding First Strand Master Mix (Invitrogen). First-strand cDNA was generated using random primers reverse transcription, followed by second-strand cDNA synthesis. The synthesized cDNA was subjected to end-repair and then was 3′ adenylated. Adapters were ligated to the ends of these 3′ adenylated cDNA fragments. Several rounds of PCR amplification with PCR Primer Cocktail and PCR Master Mix were performed to enrich the cDNA fragments. Then, the PCR products are purified with Ampure XP Beads. The final library was analyzed using two methods: checking the distribution of the fragment size using the Agilent 2100 bioanalyzer and quantifying the library using real-time quantitative PCR (QPCR) (TaqMan Probe). The Qualified libraries were sequenced pair end on the Hiseq 4000 or Hiseq X-ten platform (BGI-Shenzhen, China).

MicroRNA library was constructed with 1 μg total RNA for each sample. The total RNA was purified on a 15% urea denaturing polyacrylamide gel electrophoresis (PAGE) by electrophoretic separation; then, small RNA regions were excised and recovered, corresponding to the 18–30 nt bands in the marker lane (14–30 ssRNA Ladder Marker, TAKARA). Afterward, small RNAs of the 18–30 nt were ligated to a 5′-adaptor and a 3′- adaptor. The small RNA ligated with adapters was then transcribed by SuperScript II Reverse Transcriptase (Invitrogen, United States) into cDNA. PCR Primer Cocktail and PCR Mix were then used to accomplish rounds of PCR amplification. The PCR products were screened through agarose gel electrophoresis with target fragments 100–120 bp and purified using QIAquick Gel Extraction Kit (QIAGEN, Valencia, CA, United States). Two methods were applied to quality and quantitate the library: using the Agilent 2100 bioanalyzer to inspect the distribution of the size of the fragments; employing real-time quantitative PCR (q-PCR) (TaqMan Probe) to quantify the library. The final ligation PCR products were subsequently sequenced with the BGIseq-500 platform (BGI).

Circular RNA library was constructed with 3 μg of total RNA treated with DNase I for degrading double and single-stranded DNA presenting in RNA samples. The Ribo-off rRNA Depletion Kit (Vazyme, Inc.) was used to remove ribosomal RNA rather than purifying poly-A RNA using poly dT primer beads. Linear RNA was removed by RNase R (Epicentre, lnc). Agencourt RNAClean XP magnetic beads were used to accomplish the purification. All other steps were executed according to the manual instruction. Two methods were applied to quality and quantitate the library: using the Agilent 2100 bioanalyzer to inspect the distribution of the fragments size, and employing BMG (OMEGA) to quantify the library. Finally, the qualified libraries were subsequently sequenced pair end on the BGIseq-500 (BGI).

The sequencing data were filtered using SOAPnuke (v1.5.2) (Li et al., 2008) by Dewan et al. (2018) removing reads that contain sequencing adapter; (Pundlik et al., 2020) removing reads that have low-quality base ratio (base quality less than or equal to 5) over 20%; (Maas et al., 2017) removing reads with unknown base (’ N’ base) ratio over 5%, following which the clean reads were obtained and stored in a format of FASTQ. HISAT2 (v2.0.4) (Kim et al., 2015) was employed to map the clean reads to the reference genome. The clean reads were aligned to the reference coding gene set using Bowtie2 (v2.2.5) (Langmead and Salzberg, 2012), after which the expression level of a gene was calculated by RSEM (v1.2.12) (Li and Dewey, 2011).

The experimental flow chart was created with BioRender.com. The heatmap volcano and MA plot were plotted by the R package (pheatmap and ggplot2). The linear counterparts of circRNA were obtained from the circBase^2^ (Glažar et al., 2014).

### Real-time quantitative PCR

Employing the RNA Extraction Kit (Bio-Tek, Winooski, VT, United States) to extract total RNA, and mixed it with the reaction solution conFig.d with reverse transcription reagent (RT Master Mix for qPCR Kit, MedChemExpress, Monmouth Junction, NJ, United States), then put it into gradient PCR instrument for reverse transcription reaction. A total of 3 U/mg RNase R (Epicentre, Madison, WI, United States) was used to perform the RNase R treatment for 15 min at 37°C. The cDNA formed by reverse transcription was then conFig.d with SYBR green (SYBR^®^ Green qPCR Master Mix, MedChemExpress, Monmouth Junction, NJ, United States). The primers for mmu-miR-1247-5p, mmu-miR-214-3p and mmu-miR-199a-5p were purchased from RiboBio (China), The primers sequences for the detection of DEmRNAs (Krt80, Hba-a1, and Htr2a), DElncRNAs (lncRmst, lncC730002L08Rik, and lncGm10635), DEmiRNAs (mmu-miR-1247-5p, mmu-miR-214-3p and mmu-miR-199a-5p), and DEcircRNAs (mmu_circ_0014855, mmu_circ_0001104, and mmu_circ_0000494) and the GAPDH were shown in Table 1.

**TABLE 1:** Quantitative real-time polymerase chain reaction (qRT-PCR) primer sequences.

Target	Sequence (5′→ 3′)
Krt80	F: GTGAAGGCCCAGTATGACGC R: CTGCTTTGGAGGCTGTTCCC
Hba-a1	F: ATGGAGCTGAAGCCCTGGAA R: GAGCATCGGCGACCTTCTTG
Htr2a	F: TCCATCCACAGAGAGCCAGG R: AAGAACACGATGCCCAGCAC
lncRmst	F: GGTTGATGGAGTCAGGGACG R: GGCACCTGTAGAAACAGCCT
lncC730002L08Rik	F: TCATACCTCCTCTCCGTGGT R: CACTGAAATGATGCGCTGGC
lncGm10635	F: ACATGGTGCCTGCTCTCTTG R: AGTGGGGTCTTTGCGACATC
mmu_circ_0014855	F: GACAGTGAGAGCGGACAGAAT R: GCTTCGTTTTCCGTATCCGC
mmu_circ_0001104	F: TGGGTGTTAATCAGCATCTATCA R: AACATGGCGACTTCCGACTT
mmu_circ_0000494	F: GCCAGCTTGCTTGACCACATA R: TTCAGTGATTTTCCGAGGCCG
mmu-miR-1247-5p	AACCCGTCCCGTTCGTCCC (Tailing Reaction)
mmu-miR-214-3p	AGCAGGCACAGACAGGCAGT (Tailing Reaction)
mmu-miR-199a-5p	AACCCAGTGTTCAGACTACCT (Tailing Reaction)
GAPDH (mouse)	F: AGGTCGGTGTGAACGGATTTG R: TGTAGACCATGTAGTTGAGGTCA

### Kyoto encyclopedia of genes and genomes and gene ontology analysis

Kyoto encyclopedia of genes and genomes and GO analyses were performed based on the DEmRNAs, target genes of predicted RBPs of DElncRNAs, predicted targets of DEmiRNAs, and the host gene of DEcircRNAs. KEGG analysis^3^ was conducted to obtain pathway clusters and molecular interaction. The enrichment score was denoted by -log10 (*p*-value), which demonstrates the significance of correlations of the pathway. The *p*-value was corrected by FDR. GO analysis^4^ covered the terms biological process (BP), cellular component (CC), and molecular function (MF), showing the gene annotation gene function in various organisms. The -log10 (*p*-value) denotes the enrichment score, indicating the significance of GO term enrichment. The results were visualized by the R package (ggplot2).

### Construction of the long non-coding RNA-RNA-binding proteins network

Differentially expressed lncRNA were searched in starBase database^5^ (Li et al., 2014) to find the target RNA-binding proteins. Cytoscape software (v3.9.0) was used to construct and visually display. Followed Venn diagram was plotted by http://www.bioinformatics.com.cn/.

### Long non-coding RNA-mRNA co-expression analysis

Differentially expressed lncRNAs and mRNAs were used to construct co-expression networks. The lncRNA-mRNA networks were constructed according to the signal intensity of specific expression of mRNA and lncRNA determined in the microarray analysis. For each mRNA-lncRNA pair, Pearson correlation coefficients (PCC) were calculated, and significant correlation pairs (|cor| > 0.95) were selected to construct the network. The circos heatmap was plotted using the OmicShare tools, a free online platform for data analysis.^6^

### Competing endogenous RNA network

The competing endogenous RNA mechanism between the circRNA, lncRNA, and mRNA can indirectly regulate the gene expression, and the miRNA serves as the sponge in their communication. Based on the mechanism of ceRNA, the circRNAs-miRNAs-mRNAs, and the lncRNAs-miRNAs-mRNAs network were constructed. Targetscan (v7.2)^7^ (Agarwal et al., 2015) was used to predict the target mRNA of miRNA. Predicting the downstream target miRNA of circRNA using RNAhybrid algorithm^8^ (Krüger and Rehmsmeier, 2006).

### Immunofluorescence

Brain slices (ipsilesional cortex) were rewarmed for 20 min, followed by blocking using pre-prepared blocking solution (blocking solution: goat serum = 9:1) for 1 h, and incubated with primary antibodies (1:200) overnight. TBTS was used to rinse the cells for 2 × 5 min on the following day; the secondary antibodies were incubated at 37°C for 1 h, followed by rinsing again in TBTS for 2 × 5 min. The nuclei were visualized using a DAPI-contained mounting medium. A fluorescence microscope was employed for analysis. The antibodies were SNAP-25 antibody (ZEN BIO, cat# R27294, China), VAMP-2 (ZEN BIO, cat# R27417, China), and NeuN antibody (1:200, cat# 66836-1-Ig, Protein, China).

### Western blotting

Before resolving on SDS-PAGE, proteins were extracted from the mouse ipsilesional cortex and were added to the tissue lysate for lysis, then homogenized with a homogenizer. The proteins were transferred to PVDF membranes and then incubated with the primary antibodies at 4°C overnight. After TBST washing of PVDF membrane, incubating secondary antibody at room temperature for 1 h. Image J software (1.53) was used to analyze the gray value. The β-actin (GeneTex, Cat#GTX109639, United States) was used as an internal control. Other major antibodies have been involved above.

### Statistical analysis

All the statistical analysis was determined by applying the Prism 9 (version 9.1.1). The normality test was performed. The experimental datasets were analyzed with one-way ANOVA, followed by *post-hoc* Tukey’s test. A *p*-value < 0.05 was considered to be statistically significant. For sequencing data, DESeq2(v1.4.5) (Love et al., 2014) was employed to analyze the differentially expressed non-coding and coding genes, with *Q*-value ≤ 0.05 and the | log2fold change| ≥ 1 as the screening threshold.

## Results

### Melatonin treatment reduces traumatic brain injury-induced neurological deficit

**FIGURE 1:**
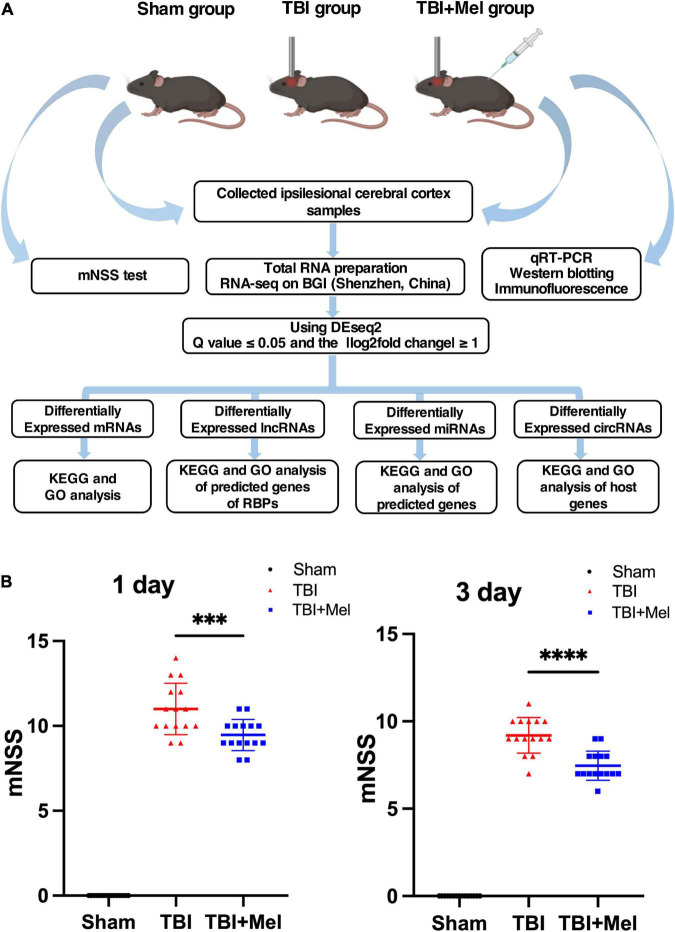
Experimental design scheme and the neural function assessment. **(A)** Study flow chart. **(B)** The neurological function of mice was assessed by the mNSS; the data were presented using mean ± SD, ***p < 0.001 TBI + Mel vs. TBI, and ****p < 0.0001 TBI + Mel vs. TBI, *n* = 15, one-way ANOVA followed by Tukey’s test.

In the present study, we performed the mNSS test to observe whether Mel could improve the neural disorder caused by TBI. Followed up analysis scheme and *in vivo* experiment are shown in Figure 1A. The mNSS scores increased in the TBI group, suggesting that the CCI model was successfully established. Compared with the TBI group, the mNSS score of the TBI + Mel group decreased at 2 time points (*p* < 0.001 and *p* < 0.0001) (Figure 1B). The above results indicated that melatonin could significantly improve neural function in mice with TBI.

It is known that the mechanism of secondary brain injury is of concern because it is more relevant for clinical translation. Research has pointed out that the pathophysiological process after TBI reached at peak on day 3 (Donkin and Vink, 2010). Consequently, we chose to perform RNA-seq on day 3 after trauma.

### Differentially expressed mRNAs, long non-coding RNAs, and circular RNAs post-traumatic brain injury

**FIGURE 2:**
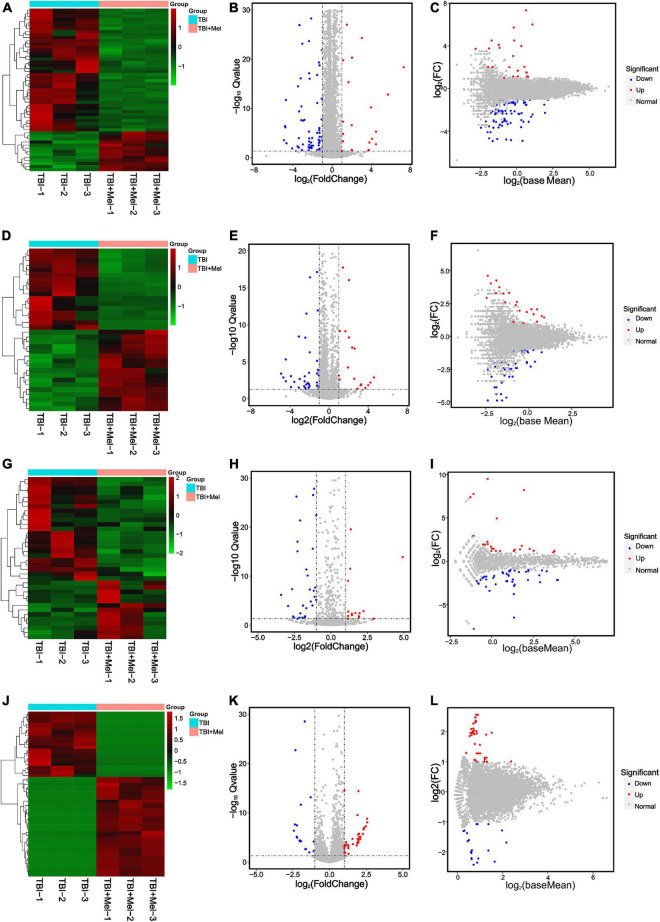
RNA-seq reveals different expression patterns of mRNAs, lncRNAs, miRNAs, and circRNAs in TBI and TBI + Mel group mice samples at 3 days. **(A–C)** Clustering analysis heatmap (plotted by selected DEmRNAs that expression level is relatively stable within groups), volcano plot and MA plot of DEmRNAs between TBI and TBI + Mel group. TBI-1, TBI-2, and TBI-3 represent three repetitions of untreated brain injury mice. TBI + Mel-1, TBI + Mel-2, and TBI + Mel-3 represent three repetitions of TBI mice treated with melatonin. **(D–F)** Clustering analysis heatmap (plotted by selected DElncRNAs that expression level is relatively stable within groups), volcano plot and MA plot of DElncRNAs between TBI and TBI + Mel group. **(G–I)** Clustering analysis heatmap (plotted by selected DEmiRNAs that expression level is relatively stable within groups), volcano plot and MA plot of DEmiRNAs between TBI and TBI + Mel group. **(J–L)** Clustering analysis heatmap (plotted by selected DEcircRNAs that expression level is relatively stable within groups), volcano plot and MA plot of DEcircRNAs between TBI and TBI + Mel group.

RNA-sequencing on cDNA and sRNA libraries was performed on the injured hemicerebrum samples from TBI groups and TBI + Mel groups after 3 days of TBI. All of our RNA-seq data were already uploaded to NCBI (Accession: PRJNA725662). Furthermore, all read counts and the clean-read ratio of sequencing data are shown in Table 2. The DEseq2 was used to detect the differential expressed coding genes and non-coding genes; significant difference was defined as fold change ≥1 and *Q*-value < 0.05. There were 93 DEmRNAs (79 down-regulation and 14 up-regulation), 48 DElncRNAs (30 down-regulation and 18 up-regulation), 59 DEmiRNAs (43 down-regulation and 16 up-regulation) and 59 DEcircRNAs (23 down-regulation and 36 up-regulation) in TBI + Mel group compared to TBI group, respectively. Figure 2 shows the clustering heatmap, volcano plot, and MA plot of DEmRNAs (Figures 2A–C), DElncRNAs (Figures 2D–F), DEmiRNAs (Figures 2G–I), and DEcircRNAs (Figures 2J–L) between the TBI group and TBI treated with the melatonin group.

**TABLE 2:** Information of quality control of RNA sequencing.

Sample	Total raw reads (M)	Total clean reads (M)	Total clean bases (Gb)	Clean reads Q20 (%)	Clean reads Q30 (%)	Clean reads ratio (%)
TBI-1	114.94	113.84	11.38	98.43	95.25	99.04
TBI-2	112.44	111.37	11.14	98.18	94.78	99.04
TBI-3	112.44	111.43	11.14	98.43	95.23	99.1
TBI + Mel-1	114.94	113.93	11.39	98.34	94.97	99.12
TBI + Mel-2	114.94	113.79	11.38	98.44	95.28	99
TBI + Mel-3	114.94	113.86	11.39	98.33	94.94	99.06

### Validation of the dysregulated non-coding RNAs and mRNAs

**FIGURE 3:**
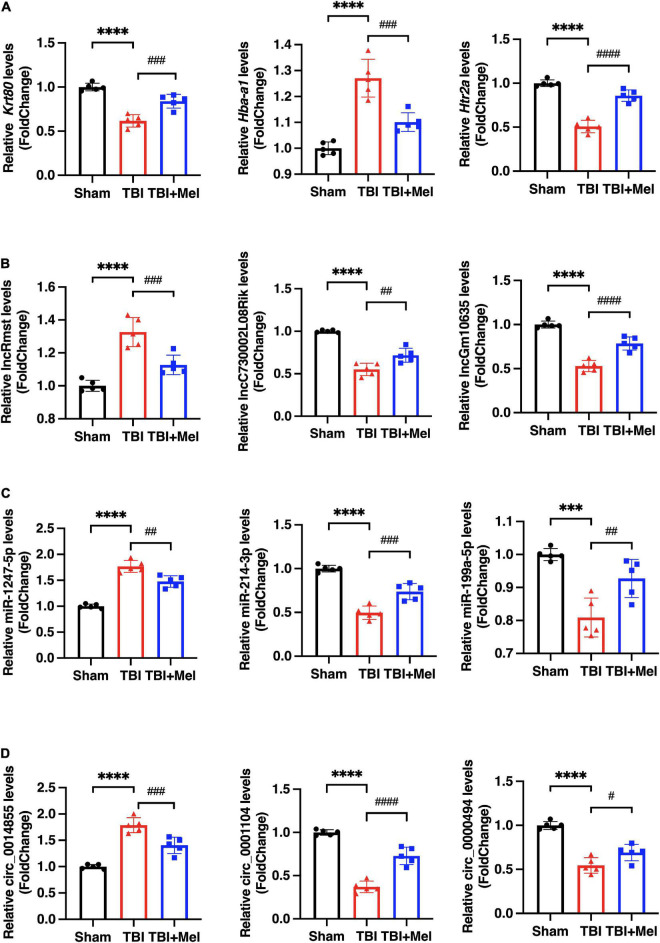
The differential expression of DEmRNAs, DElncRNAs, DEmiRNAs, and DEcircRNAs were validated by qRT-PCR. **(A)** Representative differentially expressed mRNAs (DEmRNAs), Krt80, Hba-a1, and Htr2a, were detected by qPCR. **(B)** Representative differentially expressed lncRNAs (DElncRNAs), lncRmst, lncC730002L08Rik, and lncGm10635, were detected by qPCR. **(C)** Representative differentially expressed miRNAs (DEmiRNAs), miR-1247-5p, miR-214-3p, and miR-199a-5p, were detected by qPCR. **(D)** Representative differentially expressed circRNAs (DEcircRNAs), circ_0014855, circ_0001104, and circ_0000494, were detected by qPCR. The data were presented using mean ± SD, ***p < 0.001, and ****p < 0.0001 TBI vs. Sham; ^#^*p* < 0.05, ^##^*p* < 0.01, ^###^*p* < 0.001, and ^####^*p* < 0.0001 TBI + Mel vs. TBI, one-way ANOVA followed by Tukey’s test.

To randomly choose 3 mRNAs, 3 lncRNAs, 3 miRNAs, and 3 circRNAs with differential expression to verify our RNA-seq reliability *via* qRT-PCR. As shown in Figure 3, the consequences of RNA sequencing data are consistent with qRT-PCR. Hba-a1, lncRmst, miR-1247-5p, and circ_0014855, which are randomly selected mRNAs, lncRNA, miRNA, and circRNA, respectively, were down-regulated after using Mel compared with the TBI group. Moreover, the DEmRNAs, Krt80 and Htr2a, the DElncRNAs, lncC730002L08Rik, and lncGm10635, the DEmiRNAs, miR-214-3p and miR-199a-5p, and the DEcircRNAs, circ_0001104 and circ_0000494, which expression were up-regulated in TBI + Mel group. Therefore, these further proved the accuracy of our sequencing results.

### Differentially expressed mRNAs were analyzed by Kyoto encyclopedia of genes and genomes and gene ontology analysis

**FIGURE 4:**
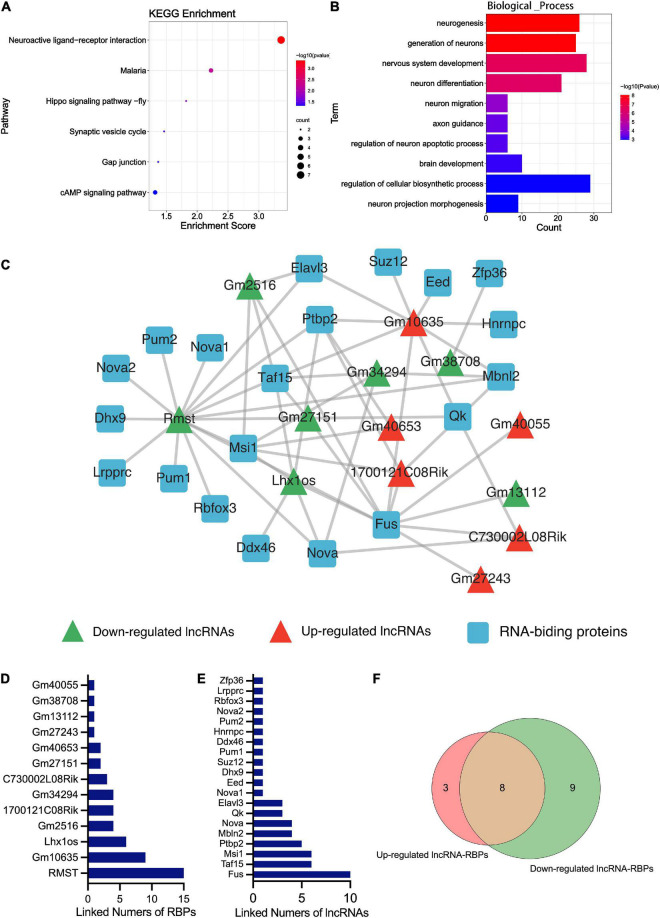
Bioinformatics analysis results of the DEmRNA and DElncRNA. **(A)** The KEGG enrichment analysis of the DEmRNAs. The abscissa enrichment score represents the enriched extent of the pathway, and the ordinate represents each pathway. The size of the dots represents the number of genes in each pathway and the color of the dots represents the -log10 (*p*-value); *p*-value was corrected by FDR. **(B)** The GO_BP enrichment analysis of the DEmRNA. The ontology only covered biological processes. The abscissa represents the count in the GO_BP term, the ordinate represents the GO_BP term, and the color of the column represents the -log10 (*p*-value). **(C)** The lncRNA-RBPs regulatory networks. Green and red triangles represent the down-regulated and up-regulated DElncRNAs, respectively. The blue round rectangle represents the RNA-binding proteins. **(D)** The numbers of lncRNAs that connected RBPs. The abscissa represents the numbers of linked RBPs and the ordinate represents the lncRNAs. **(E)** The numbers of RBPs that connected lncRNAs. The abscissa represents the numbers of linked lncRNAs and the ordinate represents the RBPs. **(F)** Venn diagram showing the overlap number of RBPs of up-regulated and down-regulated lncRNAs.

Kyoto encyclopedia of genes and genomes pathway enrichment analysis and GO enrichment analysis were performed. The KEGG analysis of total differentially expressed mRNAs (including up and down-regulated genes) showed that the enriched pathway was the neuroactive ligand-receptor interaction, followed by the synaptic vesicle cycle and the cAMP signaling pathway (Figure 4A). No study indicated the relation between malaria or hippo signaling pathway-fly and TBI. The enriched GO_BP terms of the dysregulated genes included neurogenesis, generation of neurons, nervous system development, neuron differentiation, neuron migration, axon guidance, etc. (Figure 4B). Other terms of GO analysis including GO_CC and GO_MF of DEmRNAs were shown in Supplementary Figure 1A.

### Construction of long non-coding RNA-RNA-binding proteins network and function prediction

**FIGURE 5:**
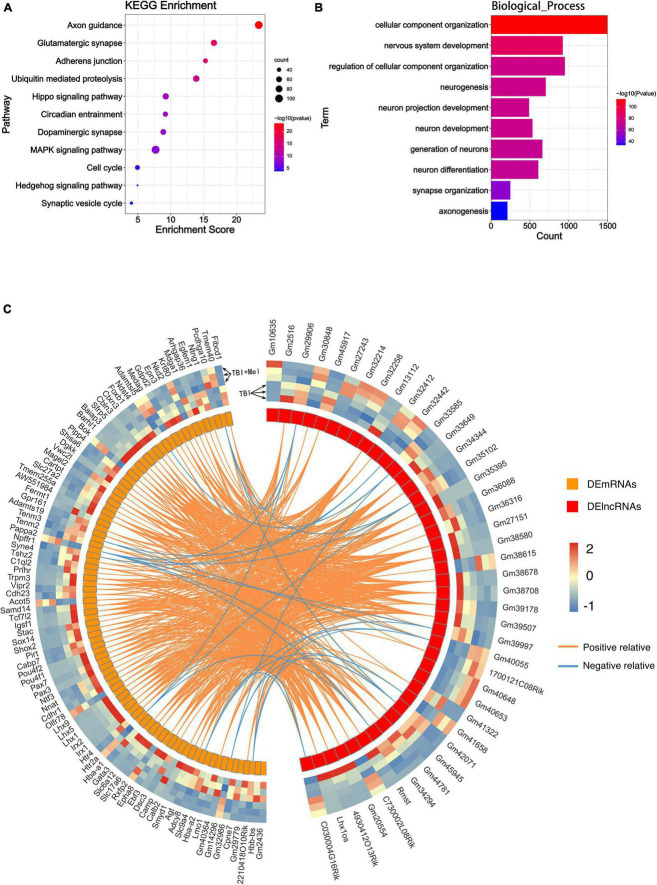
Function predicting of the DElncRNA and their correlation with DEmRNA. **(A)** The KEGG enrichment analysis of the target mRNA of RBPs of DElncRNAs. The abscissa enrichment score represents the enriched extent of the pathway, and the ordinate represents each pathway. The size of the dots represents the number of genes in each pathway, the color of the dots represents the -log10 (*p*-value), and the *p*-value was corrected by FDR. **(B)** The GO_BP enrichment analysis of the target mRNA of RBPs of DElncRNAs. The ontology only covered biological processes. The abscissa represents the count in the GO_BP term, the ordinate represents the GO_BP term, and the color of the column represents the -log10 (*p*-value). **(C)** Circos heatmap shows the correlation coefficient of DElncRNAs and DEmRNAs. The left outermost layer of the circos heatmap is the gene name of DEmRNAs and the right part is the gene name of DElncRNAs. The largest inner circles show the differentially expressed abundance; the left part is DEmRNAs; the right part is the DElncRNAs. The larger inner circles with modules represent each DElncRNA and DEmRNA. The curve inside of the circle represents the relevance between lncRNA and mRNA. Orange and blue curves represent positive and negative correlations, respectively.

RNA-binding proteins (RBPs) act as a kind of key regulator of gene expression, where lncRNAs alter gene expression to trigger or inhibit some biological process *via* interaction with RBPs (Zhang et al., 2017). In order to explore the mechanism of these lncRNAs in TBI administrated with melatonin, the starBase was used to predict the RBPs target of differentially expressed lncRNAs (lncRNAs targeting RBPs that could not be found in starBase were excluded). Figure 4C shows the predicted network of lncRNA-RBPs. We noticed that the lncRmst was the lncRNA that interacted with the most RBPs (up to 15), and Fus, one of the RBPs, was correlated with 10 lncRNAs (Figures 4D,E). Furthermore, we observed crossover of RBPs between the ascending and descending lncRNAs, after which we obtained 8 RBPs that were jointly regulated (Figure 4F). For predicting the possible function of these DElncRNAs, KEGG and GO analyses based on the predicted target genes of these RBPs were performed. The KEGG consequences have shown that the axon guidance, glutamatergic synapse, and adherens junction were the top 3 enriched pathways, and the hippo signaling pathway was also enriched (Figure 5A). Interestingly, the synaptic vesicle cycle was also enriched in KEGG of DElncRNAs. The GO_BP results revealed enrichment in the cellular component organization, nervous system development, and neurogenesis (Figure 5B). The GO_CC and GO_MF of DElncRNAs were involved in Supplementary Figure 1B.

### Long non-coding RNA-mRNA co-expression networks analysis

**FIGURE 6:**
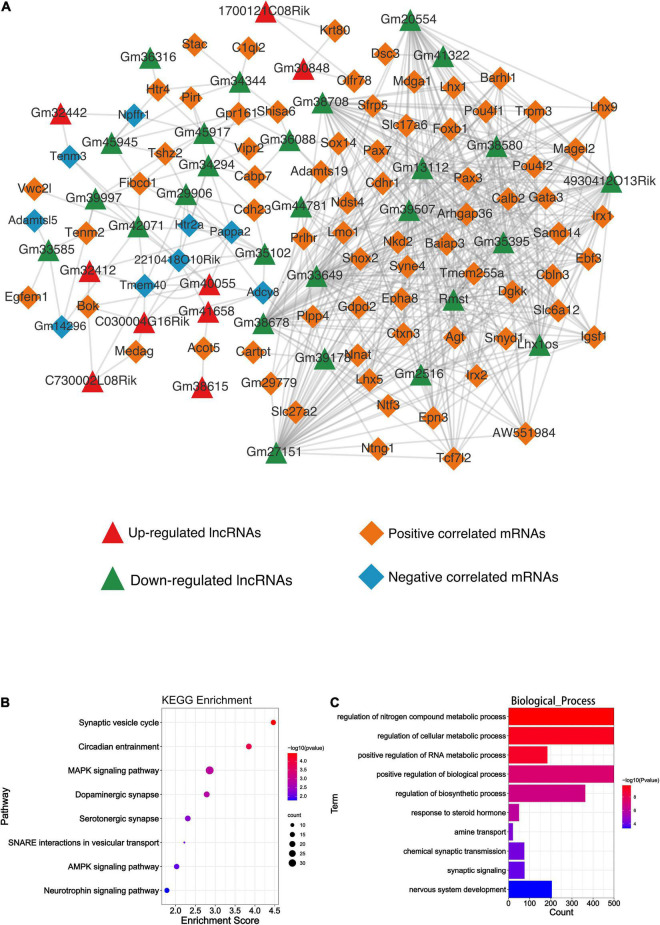
Bioinformatics analysis results of the DElncRNAs and DEmiRNAs. **(A)** The lncRNA-mRNA co-expression network. Green and red triangles represent the down-regulated and up-regulated DElncRNAs, respectively. Orange and blue diamonds represent the positive and negative correlated mRNAs, respectively. **(B)** The KEGG enrichment analysis of the predicted genes of DEmiRNAs. The abscissa enrichment score represents the enriched extent of the pathway and the ordinate represents each pathway. The size of the dots represents the number of genes in each pathway, the color of the dots represents the -log10 (*p*-value), and *p*-value was corrected by FDR. **(C)** The GO_BP enrichment analysis of the predicted genes of DEmiRNAs. The abscissa represents the count in the GO_BP term, the ordinate represents the GO_BP term, and the color of the column represents the -log10 (*p*-value).

Differentially expressed lncRNAs and DEmRNAs expression data were used to obtain the expression correlation *via* the Pearson correlation test. The positive or negative relationships between the DElncRNAs and DEmRNAs are shown in Figure 5C; all the PCC of lncRNA-mRNA pairs were included in Supplementary Table 2. The lncRNA-mRNA co-expression network was then constructed based on the statistical data (Figure 6A). Five hundred thirty-four lncRNA-mRNA pairs were involved in the network. Among all co-expression pairs contained in the network, 41 pairs were located on the same chromosome, while 493 pairs were on a different chromosome. Briefly, *cis*-regulation means lncRNA can directly regulate the expression of other neighboring genes on the same chromosome. *Trans*-acting refers to the lncRNA that can affect other genes located on different chromosomes (Kopp and Mendell, 2018). Based on our analysis, we found that DElncRNAs function more through *trans*-regulation than *cis*-regulation (Supplementary Tables 3, 4), which was consistent with Wang et al. (2018).

### Function prediction of differentially expressed microRNAs

A total of 59 DEmiRNA, including 43 down-regulated and 16 up-regulated miRNAs, were detected. Finally, 9 up-regulated and 34 down-regulated miRNAs were predicted to their downstream mRNA target through Targetscan (v7.2). All of those predicted targets were included in the KEGG and GO analysis to implicate the potential function of DEmiRNAs. As shown in Figure 6B, the synaptic vesicle cycle and circadian entrainment were mostly enriched based on the KEGG analysis, while regulation of nitrogen compound metabolic process was highly enriched based on GO_BP analysis (Figure 6C). GO_CC and GO_MF analyses of DEmiRNAs are shown in Supplementary Figure 2A.

### Function prediction of differentially expressed circular RNAs

**FIGURE 7:**
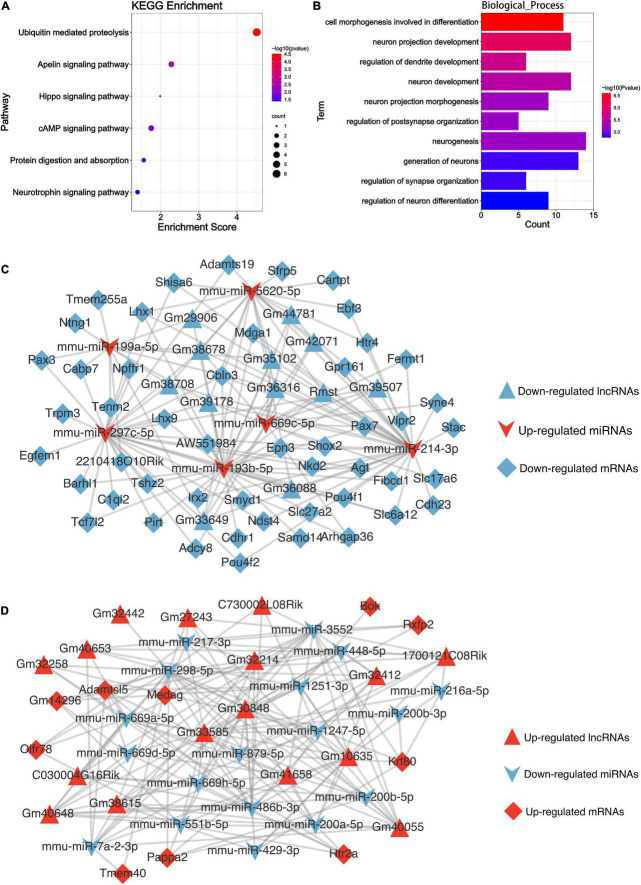
Bioinformatics analysis results of the DEncRNAs. **(A)** The KEGG enrichment analysis of the parental genes of DEcircRNAs. The abscissa enrichment score represents the enriched extent of the pathway, and the ordinate represents each pathway. The size of the dots represents the number of genes in each pathway, the color of the dots represents the -log10 (*p*-value), and the *p*-value was corrected by FDR. **(B)** The GO_BP enrichment analysis of the parental genes of DEcircRNAs. The abscissa represents the count in the GO_BP term, the ordinate represents the GO_BP term, and the column’s color represents the -log10 (*p*-value). **(C)** The competing endogenous RNA network of the descending DElncRNAs. **(D)** The competing endogenous RNA network of the ascending DElncRNAs. Triangle, V shape, and diamond represent the lncRNA, miRNA, and mRNA, respectively. Red represents up-regulated, and blue is down-regulated.

Circular RNAs can affect the splicing or transcription of host genes (Chen, 2016). To explore whether the differentially expressed circRNAs are involved in the Mel effect in TBI, we identified their linear counterparts in circbase, and then performed KEGG and GO analyses. The results of host gene-based KEGG analysis showed the enrichment of the ubiquitin-mediated proteolysis and hippo signaling pathway (Figure 7A). Cell morphogenesis involved in differentiation, neuron projection development, and regulation of dendrite development were the top 3 biological processes enriched in GO_BP analysis (Figure 7B). Other GO terms were exhibited in Supplementary Figure 2B.

### Regulatory networks of competing endogenous RNAs

**FIGURE 8:**
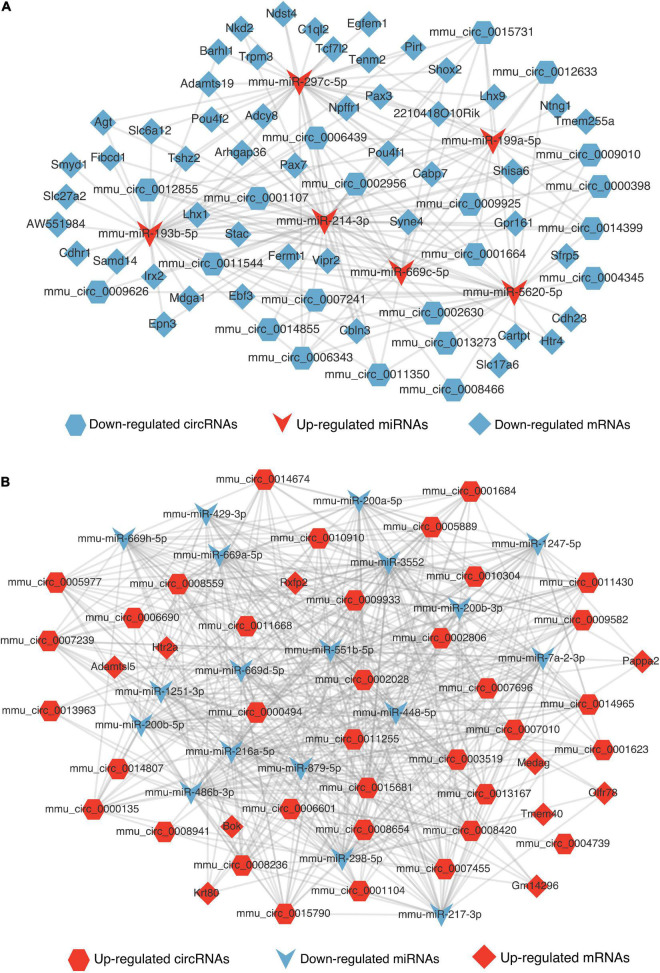
The competing endogenous RNA networks of DEcricRNA. **(A)** The competing endogenous RNA network of the decreased DEcircRNAs. **(B)** The competing endogenous RNA network of the increased DEcircRNAs. Hexagon, V shape, and diamond represent the circRNA, miRNA, and mRNA, respectively. Red represents up-regulated and blue represents down-regulated.

MicroRNAs could serve as a molecular sponge and can bind circRNAs, lncRNAs, and mRNAs, influencing gene expression; this mechanism is also known as a competing endogenous RNA mechanism. According to the hypothesis, we constructed the DElncRNA-DEmiRNA-DEmRNA (Figures 7C,D) and DEcircRNA-DEmiRNA-DEmRNA network (Figures 8A,B) using Cytoscape software (version 3.9.0) to explicit the regulatory relationship among them. Different RNA types and regulatory relations were, respectively, represented by various shapes and colors.

### Melatonin protects synaptic function after traumatic brain injury

**FIGURE 9:**
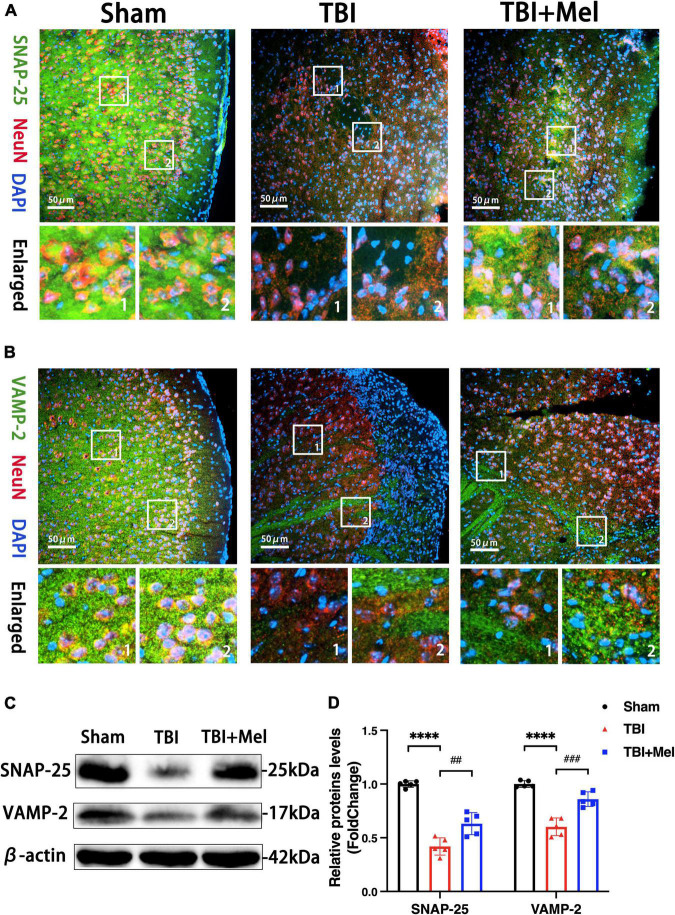
Melatonin improves the synaptic function after TBI. **(A,B)** The distributions of SNAP-25 and VAMP-2 were detected by immunofluorescent staining. The scale bar represents 50 μm. **(C,D)** The expressions of SNAP-25 and VAMP-2 in the sham, TBI, and TBI + Mel groups were detected by Western blot. *n* = 5 mice per group. SNAP-25: ****p < 0.0001 TBI vs. Sham; ^##^*p* < 0.01 TBI + Mel vs. TBI, one-way ANOVA followed by Tukey’s test. VAMP-2: ****p < 0.0001 TBI vs. Sham; ^###^*p* < 0.001 TBI + Mel vs. TBI, one-way ANOVA followed by Tukey’s test.

The synaptic vesicle cycle pathway was enriched in the KEGG analysis of differentially expressed mRNAs, lncRNAs, and miRNAs. To further verify the consequences of KEGG enrichment, we found the hub genes of this pathway using cytoHubba (Supplementary Table 5) and chose SNAP-25 and VAMP-2 to perform Western blotting and immunofluorescence to prove the results’ reliability. Our present study verified the protective effect of the synaptic function of melatonin after TBI. Firstly, the double immunofluorescence showed the SNAP-25 and VAMP-2 colocalized with NeuN, moreover, the Mel could reverse their down-regulation after TBI (Figures 9A,B). Secondly, we identified the expression of SNAP-25 and VAMP-2 after administrating Mel. Mel-treatment significantly prevented damage to the SNARE complex by increasing the protein expression of SNAP-25 and VAMP-2 compared with that in the TBI group (Figures 9C,D).

## Discussion

The exact mechanism of the protective function of Mel in TBI is unclear at present. To the best of our knowledge, this is the first study that integrated analysis of the mRNA, lncRNA, miRNA, and circRNA to uncover the potential pathway of drug action regarding the protection of melatonin in TBI. In total, 209 differentially expressed RNAs were detected. Several DEmRNAs and DEmiRNAs were reported to be correlated with TBI (Buriticá et al., 2009; Colak et al., 2012; Harper et al., 2020; Raikwar et al., 2021). Yet, the majority of ncRNAs were novel and not reported before. In KEGG and GO analyses, we have found several crucial pathways such as neuroactive ligand-receptor interaction, synaptic vesicle, and ubiquitin-mediated proteolysis, that might play a significant role in melatonin mediated anti-TBI process. Additionally, the lncRNA-RBPs network was constructed to show the interaction relationship, and we supposed that the interaction of lncRmst and Fus might be the vital mechanism of Mel in treating TBI. Furthermore, the competing endogenous RNAs networks of DElncRNAs and DEcircRNAs were constructed. Finally, we observed that the synaptic vesicle is the repeated KEGG enrichment results of DEmRNAs, DElncRNAs, and DEmiRNAs. The expression level of the hub genes of the pathway, SNAP-25, and VAMP-2, increased after using melatonin.

The positive role of Mel in TBI was illustrated through the functional analysis of the differentially expressed coding genes. Some DEmRNAs are involved in the TBI process. For example, dysregulation of *Calb2* (Buriticá et al., 2009), *Hba-a1* (Harper et al., 2020), and *C1ql2* (Colak et al., 2012) have a role in various caused TBI courses. GO_BP analyses showed that the DEmRNAs enriched in neurogenesis, generation of neurons, and nervous system development. A previous study indicated that neural function could be ameliorated by improving endogenous neurogenesis through melatonin (Chern et al., 2012). Our results further confirm the protective role of Mel in the nervous system. The neuroactive ligand-receptor interaction was the significantly enriched pathway in KEGG analysis. *Htr2a*, also known as *5-Ht2a*, is an enriched gene in that pathway. Previous research has reported that the agonist of the *5-Ht2a* receptor could diminish neuronal apoptosis in the hippocampus (Shahidi et al., 2019). In our study, *Htr2a* was up-regulated in Mel-administrated samples, which further suggests that *Htr2a* might be a potential drug target of melatonin-mediated anti-apoptotic action in TBI.

The differentially expressed ncRNAs were focused, including DElncRNAs, DEmiRNAs, and DEcircRNAs. The lncRNA was reported that could regulate the expression of target genes through RBPs, which act as molecular sponges (Lee et al., 2016). In this study, we used starBase to predict the RBPs which might interact with those DElncRNAs. LncRmst has been associated with several ce-RNA axes, including lncRmst/miR-204-5p/VCAM1 (Yin et al., 2021) and lncRmst/miR-107/Bcl2l2 (Cheng et al., 2020), have been associated with ischemic brain injury. To knockdown of the lncRmst could improve the progression of IS, attenuate OGD model injury, and regulate neuronal apoptosis (Cheng et al., 2020). In this study, we found a decrease in lncRmst after treatment with Mel; in addition, our lncRNA-RBPs network showed it has an interaction with Fus, one of the RBPs. The relationship between lncRmst and the Fus has already been reported by Liu et al. (2020). Thus, our consequences suggested that lncRmst might be a new drug target of melatonin against TBI through action on RNA-binding proteins.

For predicting the function of DElncRNAs, we overlapped the RBPs of up and down-regulated lncRNAs and searched the downstream target genes of RBPs in starBase. KEGG and GO analyses based on those predicted genes were performed. Axon guidance, glutamatergic synapse, and adherens junction were the enriched pathways based on KEGG. Axonal regeneration (Stazi et al., 2021), activating glutamatergic synaptic transmission (Evely et al., 2016), and enhancement of adherens junctions regarding barrier-protective effects (Yuan et al., 2011) have already been associated with functions of melatonin in other diseases. Interestingly, the synaptic vesicle cycle was also enriched in the results of DElncRNAs, like the enrichment of DEmRNAs; this might indicate that the synaptic vesicle cycle might have an essential role in the protective effect of Mel in TBI. Similar to KEGG, the outcomes of GO_BP mostly focused on cellular process and biological regulation. These results further confirmed the protective effect of melatonin through the DElncRNAs. Furthermore, we constructed a lncRNA-mRNA co-expression network using a previously described approach (Yang et al., 2017) to demonstrate interactions between DElncRNAs and DEmRNAs, including 36 lncRNAs and 80 mRNAs. Among 534 pairs of lncRNA-mRNA, there are 41 pairs located in the same chromosome. Chen et al. (2015) reported that the expression of *Calb2* increases significantly after ischemic brain damage, while Turner et al. (2007) suggested that *Calb2* is involved in the intrinsic neuron apoptotic pathway. *Calb2* was positively correlated with 12 down-regulated lncRNAs in our network. As overturn of the neuron apoptosis could be considered as a protective factor for TBI prognosis, the co-expression network indicated potential intrinsic regulatory relationships through *cis*- or *trans*- regulation between these DElncRNAs and DEmRNAs involved in TBI, which also might be new targets of Mel treatment.

The DEmiRNAs were involved in some pathways that have pivotal functions in Mel treating TBI. Several DEmiRNAs were reported before. MiR-200 family has been reported they might have neuroprotective effects Santra et al. (2016) and Raikwar et al. (2021) reported miR-155-5p increased after TBI. In KEGG results, based on predicted mRNA downstream, these DEmiRNAs were reported to be mostly enriched in synaptic vesicle cycle pathways. Rehman et al. (2019) demonstrated that Mel treatment could reverse the decreased synaptic protein and deregulate cognitive function caused by TBI. One other function of Mel was circadian entrainment (Claustrat et al., 2005). Additionally, we noticed that the GO_BP results were the regulation of the nitrogen compound metabolic process. Mel was reported to be able to scavenge oxygen and nitrogen-based reactants to protect mitochondria from oxidative damage (León et al., 2005). The above results indicated that the DEmiRNAs had vital functions in the Mel-mediated anti-TBI process.

The function predicting of DEcircRNAs based on the host genes indicated the essential role of the Mel after TBI. Increasing evidence indicated the circRNAs, at different regulatory levels, influenced the host gene expression (Shao et al., 2021). Xu et al. (2020) suggested that circRNA SMARCA5 could inhibit the expression of SMARCA5 by forming R-loops with the host gene, involved in DNA damage repair in cancer. Interestingly, *Tshz2* is the linear counterpart of mmu_circ_0001107, which also is a DEmRNA in our sequencing results. There is a possible implicit regulation between mmu_circ_0001107 and its host gene in Mel’s role in treating TBI, which needs to be further proved. Ubiquitin-mediated proteolysis was enriched in KEGG. Yao et al. (2006) suggested this course should be activated to obliterate the damaged proteins due to TBI and prevent pathological processes such as protein aggregation, oxidation, or apoptosis. DEcircRNAs might be crucial in the mechanism of melatonin acting on TBI through this pathway. It’s worth noting that hippo signaling pathway were enriched in the KEGG result of DElncRNAs and DEcircRNAs, Zhu et al. (2021) reported neutrophil extracellular traps (NETs) formation might be associated with sympathetic hyperactivity after TBI, and NETs could promote microglia to release IL-1βvia the Hippo/MST1 pathway. Therefore, the hippo signaling pathway might play a role in the TBI process, which needs further identification. In the BP category, the results were similar to the GO_BP results of DEmRNAs or DElncRNAs, also mainly related to cellular processes or biological regulation. These findings suggested that DEcircRNAs, similar to DElncRNAs and DEmRNAs, might also have an important effect on TBI administration with Mel by altering the processes above.

The DElncRNA-DEmiRNA-DEmRNA and DEcircRNA-DEmiRNA-DEmRNA regulatory networks exhibited the interaction of these differentially expressed ncRNAs. Recent studies have suggested an interaction network among the mRNAs, lncRNAs, and circRNAs, that communicated and regulated the mutual expression level by competing for the binding sites of miRNAs (Tay et al., 2014). Although the KEGG of circRNAs has not shown their function involving the synaptic vesicle cycle, based on our network, we assumed that DEcircRNAs might function in synapsis through the regulatory relationships. Nevertheless, the majority of the ncRNAs have not been studied yet. The regulatory relationships in our competing endogenous RNA networks need to be further verified, and they might be the potential new mechanism involving Mel treatment in TBI.

Mel could ameliorate the damage to the SNARE complex to prevent the synapsis injury in the TBI mouse model. Based on the KEGG results of DEmRNAs, DElncRNAs, and DEmiRNAs, we next found the hub genes (SNAP-25 and VAMP-2) of the synaptic vesicle cycle pathway and identified the localization and expression level. SNAP-25 and VAMP-2 are the component of the soluble N-ethylmaleimide-sensitive factor attachment protein receptor (SNARE) complex. Its formation is critical for initiating vesicle docking and plasma membrane fusion at the presynaptic terminal. Studies have shown impaired SNARE protein in the hippocampus after TBI, which may contribute to neurobehavioral dysfunction (Carlson et al., 2017a). Also, a previous study suggested that lithium could increase the expression of SNARE protein to improve neural function after TBI (Carlson et al., 2017b). In the present study, the effect of Mel in increasing the abundance of SNARE protein, SNAP-25, and VAMP-2, was preliminarily identified, thus further suggesting that preventing the loss of presynaptic protein after TBI was one of the significant mechanisms of anti-TBI process.

## Conclusion

These data offer novel insight into the molecular effect of Mel in treating TBI. They also suggest that the high-throughput sequencing and analysis of transcriptomes are useful for studying the drug mechanisms of TBI treatment.

## Data availability statement

The datasets presented in this study can be found in online repositories. The names of the repository/repositories and accession number(s) can be found in the article/Supplementary material.

## Ethics statement

The animal study was reviewed and approved by the Ethics Committee of the First Affiliated Hospital of Chongqing Medical University (No. 2021-177).

## Author contributions

JF and QZ: study design and conception. JF and BW: animal experiments and TBI model establishment. XH, WT, and ZT: bioinformatics analysis. CW, QZ, JM, and EB: statistical analysis. JF, QZ, MD, CW, and ZL: manuscript draft. All authors approved the final version of the manuscript.

## Footnotes

1
http://www.lasec.cuhk.edu.hk/sample-size-calculation.html

2
http://circrna.org/

3
http://www.genome.jp/kegg/

4
www.geneontology.org

5
http://starbase.sysu.edu.cn/

6
https://www.omicshare.com/tools/

7
http://www.targetscan.org/mmu_71/

8
https://bibiserv.cebitec.uni-bielefeld.de/rnahybrid

## Supplementary material

Supplementary Figure 1 GO cellular component and GO_MF analysis results of the DEmRNAs and DElncRNAs. **(A)** The GO_CC and GO_MF enrichment analysis of the predicted genes of DEmRNAs. The abscissa represents the count in the GO_CC and GO_MF term, the ordinate represents the GO_CC and GO_MF term, and the color of the column represents the -log10 (*p*-value). **(B)** The GO_CC and GO_MF enrichment analysis of the predicted genes of DElncRNAs. The abscissa represents the count in the GO_CC and GO_MF term, the ordinate represents the GO_CC and GO_MF term, and the color of the column represents the -log10 (*p*-value).

Supplementary Figure 2 GO cellular component and GO_MF analysis results of the DEmiRNAs and DEcircRNAs. **(A)** The GO_CC and GO_MF enrichment analysis of the predicted genes of DEmiRNAs. The abscissa represents the count in the GO_CC and GO_MF term, the ordinate represents the GO_CC and GO_MF term, and the color of the column represents the -log10 (*p*-value). **(B)** The GO_CC and GO_MF enrichment analysis of the predicted genes of DEcircRNAs. The abscissa represents the count in the GO_CC and GO_MF term, the ordinate represents the GO_CC and GO_MF term, and the color of the column represents the -log10 (*p*-value).

## Abbreviations

TBI: traumatic brain injury
Mel: melatonin
MiRNA: MicroRNA
LncRNA: long non-coding RNA
CircRNA: circular RNA
DE: differentially expressed
DEmRNAs: differentially expressed mRNAs
DElncRNAs: differentially expressed lncRNAs
DEmiRNAs: differentially expressed miRNAs
DEcircRNAs: differentially expressed circRNAs
NcRNAs: non-coding RNAs
RBPs: RNA-binding proteins
QRT-PCR: quantitative real-time polymerase chain reaction
KEGG: Kyoto encyclopedia of genes and genomes
GO: gene ontology
GO_BP: GO biological process
GO_CC: GO cellular component
GO_MF: GO molecular function
CCI: controlled cortical impact
MNSS test: modified neurological severity score test
SsCir DNA: single-strand circle DNA
DNB: DNA nanoball
PAGE: polyacrylamide gel electrophoresis
PCC: pearson correlation coefficients
IVC: individually ventilated cages.
