# Supplementary material for: Protective effects and regulatory pathways of melatonin in traumatic brain injury mice model: Transcriptomics and bioinformatics analysis
Source: Front Mol Neurosci. 2022 Sep 9;15:974060. doi: 10.3389/fnmol.2022.974060 (PMC9500234; doi:10.3389/fnmol.2022.974060)
Supplement: Supplementary file 1 [file Table_1.DOC]

**Supplementary Table 1. Modified neurological severity score**

|  | Points |
| --- | --- |
| Motor tests | 2 |
| 1 Flexion of forelimb |  |
| 1 Flexion of hindlimb |  |
| Placing rat on the floor (normal = 0; maximum = 3) | 3 |
| 0 Normal walk |  |
| 1 Inability to walk straight |  |
| 2 Circling toward the paretic side |  |
| 3 Fall down to the paretic side |  |
| Sensory test | 3 |
| 1 Placing test (visual tactile test) |  |
| 2 Proprioceptive test (deep sensation, pushing the paw against the table edge) |  |
| Beam balance test (normal = 0; maximum = 6) | 6 |
| 0 Balance with steady posture |  |
| 1 Grasps side of beam |  |
| 2 Hugs the beam and one limb falls down from the beam |  |
| 3 Hugs the beam and two limbs fall down from the beam, or spins on beam (> 60 s) |  |
| 4 Attempts to balance on the beam but falls off (> 40 s) |  |
| 5 Attempts to balance on the beam but falls off (> 20 s) |  |
| 6 Falls off: No attempt to balance or hang on the beam (< 20 s) |  |
| Reflexes absent and abnormal movements | 4 |
| 1 Corneal reflex (eye blink when lightly touching the cornea with cotton) |  |
| 1 Startle reflex (motor response to a brief noise) |  |
| 1 Auricle reflex (shaking head when touching the external auditory canal) |  |
| 1 Seizures, myoclonus, myodystony |  |
| Maximum points | 18 |
