# Supplementary material for: Protective effects and regulatory pathways of melatonin in traumatic brain injury mice model: Transcriptomics and bioinformatics analysis
Source: Front Mol Neurosci. 2022 Sep 9;15:974060. doi: 10.3389/fnmol.2022.974060 (PMC9500234; doi:10.3389/fnmol.2022.974060)
Supplement: Supplementary file 2 [file Table_2.DOCX]

| Supplementary Table 2. Pearson Correlation Coefficients | | |
| --- | --- | --- |
| mRNA | lncRNA | cor |
| Cbln3 | Gm35395 | 0.999997669 |
| Gata3 | 4930412O13Rik | 0.999957761 |
| Calb2 | Gm38580 | 0.999902459 |
| Sfrp5 | 4930412O13Rik | 0.999569885 |
| Ebf3 | 4930412O13Rik | 0.999013832 |
| Trpm3 | Gm27151 | 0.999007317 |
| Tcf7l2 | Gm20554 | 0.998995489 |
| Trpm3 | Gm13112 | 0.998856016 |
| Dgkk | Gm38580 | 0.998821134 |
| Cbln3 | Lhx1os | 0.998798459 |
| Pirt | Gm45945 | 0.998781099 |
| Trpm3 | 4930412O13Rik | 0.998709722 |
| Baiap3 | Gm38580 | 0.998706251 |
| Pou4f2 | Lhx1os | 0.998694505 |
| Pax3 | Lhx1os | 0.998606089 |
| Irx1 | Gm20554 | 0.998490048 |
| Lhx1 | Gm38580 | 0.998441327 |
| Igsf1 | Gm38580 | 0.998386203 |
| Lhx1 | Lhx1os | 0.998214325 |
| Pou4f2 | Gm38580 | 0.998152448 |
| Foxb1 | 4930412O13Rik | 0.998096413 |
| Samd14 | Gm39507 | 0.998043548 |
| Irx1 | Gm38580 | 0.997906321 |
| Barhl1 | Lhx1os | 0.997881962 |
| Trpm3 | Gm33649 | 0.997826449 |
| Prlhr | Gm38678 | 0.997818005 |
| Igsf1 | Gm20554 | 0.997776253 |
| Epn3 | Gm27151 | 0.997598964 |
| Pax3 | Gm38580 | 0.997554471 |
| Slc6a12 | Gm39507 | 0.997534602 |
| Irx2 | 4930412O13Rik | 0.99749347 |
| Slc17a6 | Gm27151 | 0.997487084 |
| Sfrp5 | Gm13112 | 0.99743001 |
| Htr4 | Gm45945 | 0.99741646 |
| Lhx5 | Gm38580 | 0.997309859 |
| Calb2 | Lhx1os | 0.997252611 |
| Lhx9 | 4930412O13Rik | 0.997226516 |
| Arhgap36 | Gm38580 | 0.997218095 |
| Gata3 | Gm27151 | 0.99720789 |
| Gata3 | Gm38678 | 0.997029149 |
| Barhl1 | Gm38580 | 0.997014561 |
| Pou4f1 | Lhx1os | 0.996988455 |
| Epn3 | 4930412O13Rik | 0.99698146 |
| Baiap3 | Gm20554 | 0.996975763 |
| Gata3 | Gm13112 | 0.996889652 |
| Lhx9 | Gm13112 | 0.996868473 |
| Epn3 | Gm33649 | 0.996777779 |
| Slc17a6 | Gm33649 | 0.996718686 |
| Dgkk | Lhx1os | 0.996539017 |
| Lhx9 | Gm38580 | 0.996429057 |
| Sfrp5 | Gm27151 | 0.996398375 |
| Tcf7l2 | Gm38580 | 0.996254605 |
| Irx2 | Gm13112 | 0.996184085 |
| Lhx5 | Lhx1os | 0.996022902 |
| Tmem255a | Gm39507 | 0.995954038 |
| Bok | C730002L08Rik | 0.995911904 |
| Ebf3 | Gm13112 | 0.995894292 |
| Magel2 | Gm33649 | 0.995702491 |
| Tenm2 | Gm33585 | 0.995618759 |
| Foxb1 | Gm38678 | 0.995534519 |
| Tcf7l2 | Gm13112 | 0.99551396 |
| Pou4f1 | Gm38580 | 0.995466753 |
| Trpm3 | Gm20554 | 0.99540684 |
| Trpm3 | Gm38678 | 0.99535749 |
| Igsf1 | Gm13112 | 0.995218204 |
| Gata3 | Gm33649 | 0.995093982 |
| Dgkk | Gm35395 | 0.995081017 |
| Pou4f2 | Gm35395 | 0.995046644 |
| Lhx5 | 4930412O13Rik | 0.995039589 |
| Slc17a6 | Gm38678 | 0.994903255 |
| Pax3 | Gm35395 | 0.99489199 |
| Magel2 | Gm20554 | 0.99485747 |
| Irx2 | Gm38580 | 0.994826982 |
| Magel2 | Gm39507 | 0.994822405 |
| Stac | Gm34344 | 0.994782886 |
| Sfrp5 | Gm38678 | 0.994662803 |
| Prlhr | 4930412O13Rik | 0.994625228 |
| Smyd1 | Gm39507 | 0.99461152 |
| AW551984 | Gm38580 | 0.994514703 |
| Sfrp5 | Gm33649 | 0.994500953 |
| Lhx5 | Gm13112 | 0.99446028 |
| Magel2 | Gm27151 | 0.994422389 |
| Tcf7l2 | Gm33649 | 0.994384596 |
| 2210418O10Rik | Gm29906 | 0.994350648 |
| Lhx9 | Gm20554 | 0.994252124 |
| Lhx1 | Gm35395 | 0.994141226 |
| Calb2 | Gm35395 | 0.994107928 |
| Lhx9 | Gm27151 | 0.993956225 |
| Ebf3 | Gm27151 | 0.993942756 |
| Barhl1 | Gm35395 | 0.993795566 |
| Arhgap36 | Gm20554 | 0.993763893 |
| Cbln3 | Gm38580 | 0.993720195 |
| Epn3 | Gm13112 | 0.99368993 |
| Slc6a12 | Gm33649 | 0.993664923 |
| Foxb1 | Gm13112 | 0.993649402 |
| Irx1 | Gm13112 | 0.993575845 |
| Ebf3 | Gm38678 | 0.993404875 |
| Tcf7l2 | Gm38708 | 0.993392089 |
| Tcf7l2 | Gm27151 | 0.993343211 |
| Prlhr | Gm27151 | 0.993142451 |
| Slc17a6 | Gm13112 | 0.993106588 |
| Pou4f1 | 4930412O13Rik | 0.993094995 |
| Krt80 | Gm30848 | 0.993051343 |
| Epn3 | Gm38678 | 0.992953399 |
| Tmem255a | Rmst | 0.992928288 |
| Calb2 | Gm20554 | 0.992910289 |
| Irx2 | Lhx1os | 0.992889523 |
| Lhx9 | Lhx1os | 0.99286696 |
| Egfem1 | Gm33585 | 0.992845775 |
| Irx2 | Gm27151 | 0.992708496 |
| Lhx9 | Gm33649 | 0.992618314 |
| Pou4f1 | Gm35395 | 0.992411556 |
| Igsf1 | 4930412O13Rik | 0.99230591 |
| Igsf1 | Lhx1os | 0.992275944 |
| Igsf1 | Gm33649 | 0.992268293 |
| Syne4 | Gm39178 | 0.992245052 |
| Sfrp5 | Gm20554 | 0.992158109 |
| Baiap3 | Lhx1os | 0.992055036 |
| Tenm2 | Gm39997 | 0.99202881 |
| Igsf1 | Gm27151 | 0.992027797 |
| Olfr78 | Gm30848 | 0.99184661 |
| Foxb1 | Gm27151 | 0.991811359 |
| Mdga1 | Gm41322 | 0.991765864 |
| Ebf3 | Gm38580 | 0.991757865 |
| Slc17a6 | 4930412O13Rik | 0.991668515 |
| Lhx1 | 4930412O13Rik | 0.991664052 |
| 2210418O10Rik | Gm42071 | 0.991659769 |
| Sfrp5 | Gm38580 | 0.991648966 |
| Ebf3 | Gm33649 | 0.991637739 |
| Irx2 | Gm38678 | 0.991627057 |
| Tmem40 | Gm40055 | 0.991618924 |
| Igsf1 | Gm38708 | 0.991556578 |
| Lhx1 | Gm13112 | 0.991554926 |
| Barhl1 | 4930412O13Rik | 0.991533484 |
| Slc6a12 | Gm27151 | 0.991486549 |
| Lhx5 | Gm20554 | 0.991414327 |
| Slc27a2 | Gm39178 | 0.991368507 |
| Tcf7l2 | 4930412O13Rik | 0.991242158 |
| Epn3 | Gm38708 | 0.991182707 |
| Epn3 | Gm20554 | 0.991151107 |
| Pirt | Gm45917 | 0.991119572 |
| Ctxn3 | Gm39507 | 0.9910931 |
| Irx2 | Gm20554 | 0.991049149 |
| Epha8 | Rmst | 0.990981222 |
| Pax3 | 4930412O13Rik | 0.990937431 |
| Irx2 | Gm33649 | 0.990931583 |
| Pou4f2 | 4930412O13Rik | 0.99082919 |
| Baiap3 | Gm13112 | 0.990649909 |
| Arhgap36 | Lhx1os | 0.990627564 |
| Magel2 | Gm13112 | 0.99061858 |
| Lhx9 | Gm38678 | 0.99061328 |
| Lhx5 | Gm35395 | 0.990508001 |
| Barhl1 | Gm13112 | 0.990376305 |
| Prlhr | Gm13112 | 0.990350997 |
| Pou4f1 | Gm13112 | 0.990324527 |
| Irx1 | Lhx1os | 0.990279979 |
| Prlhr | Gm33649 | 0.990256597 |
| Irx1 | Gm33649 | 0.99023683 |
| Trpm3 | Gm38580 | 0.990156245 |
| Lhx1 | Gm20554 | 0.990018927 |
| Pou4f2 | Gm13112 | 0.990001075 |
| Irx1 | Gm27151 | 0.989987586 |
| Plpp4 | Gm39178 | 0.989985349 |
| Dgkk | Gm20554 | 0.989922957 |
| Pax3 | Gm13112 | 0.989913935 |
| Lhx5 | Gm27151 | 0.989861931 |
| AW551984 | Gm20554 | 0.989775731 |
| Calb2 | Gm13112 | 0.989633703 |
| Slc6a12 | Gm20554 | 0.989559595 |
| Ebf3 | Lhx1os | 0.989531841 |
| Ebf3 | Gm20554 | 0.989483407 |
| Olfr78 | 1700121C08Rik | 0.989382631 |
| Gata3 | Gm20554 | 0.989360688 |
| Lmo1 | Gm44781 | 0.989299272 |
| Foxb1 | Gm33649 | 0.988942691 |
| Tshz2 | Gm34294 | 0.988785881 |
| Cdhr1 | Gm38678 | 0.9886401 |
| Irx1 | 4930412O13Rik | 0.988639485 |
| Adcy8 | Gm35102 | 0.988605471 |
| Slc6a12 | Gm13112 | 0.988601259 |
| Lhx9 | Gm38708 | 0.988570552 |
| Pou4f2 | Gm20554 | 0.988568268 |
| Tmem255a | Gm20554 | 0.988436208 |
| Gm29779 | Gm44781 | 0.988399538 |
| Lhx5 | Gm33649 | 0.988300348 |
| Shox2 | Gm38678 | 0.988262812 |
| Cartpt | Gm39178 | 0.98817813 |
| Slc6a12 | Rmst | 0.988077675 |
| Agt | Gm39507 | 0.988063796 |
| AW551984 | Lhx1os | 0.987995228 |
| Arhgap36 | Gm35395 | 0.987981349 |
| Trpm3 | Gm38708 | 0.987914511 |
| Tcf7l2 | Lhx1os | 0.987798646 |
| Baiap3 | Gm35395 | 0.987729351 |
| Pax3 | Gm20554 | 0.987507487 |
| Barhl1 | Gm20554 | 0.987499375 |
| Sfrp5 | Gm38708 | 0.987425497 |
| Sfrp5 | Lhx1os | 0.987411127 |
| 2210418O10Rik | Gm35102 | 0.987402833 |
| Lhx5 | Gm38678 | 0.987350258 |
| Adamts19 | Gm36088 | 0.987208284 |
| Slc17a6 | Gm20554 | 0.986930922 |
| Magel2 | Rmst | 0.986911466 |
| Ctxn3 | Gm33649 | 0.986874501 |
| Htr4 | Gm45917 | 0.98681583 |
| Irx1 | Rmst | 0.986779327 |
| Baiap3 | Gm33649 | 0.986686826 |
| AW551984 | Gm35395 | 0.986665096 |
| Irx2 | Gm38708 | 0.986651787 |
| Cdhr1 | Gm27151 | 0.986591345 |
| Baiap3 | Gm38708 | 0.986484203 |
| Gata3 | Gm38580 | 0.986467894 |
| Irx1 | Gm38708 | 0.986415388 |
| Igsf1 | Gm35395 | 0.986304035 |
| Calb2 | 4930412O13Rik | 0.986264892 |
| Tcf7l2 | Rmst | 0.98625093 |
| Cdhr1 | Gm33649 | 0.98622185 |
| Lhx5 | Gm38708 | 0.986220028 |
| C1ql2 | Gm34344 | 0.986212777 |
| Npffr1 | Gm45917 | 0.986206487 |
| Baiap3 | Gm27151 | 0.98603357 |
| Samd14 | Rmst | 0.985982997 |
| Lhx9 | Gm35395 | 0.985972373 |
| Irx2 | Gm35395 | 0.985943579 |
| Pou4f1 | Gm20554 | 0.985868456 |
| Baiap3 | Rmst | 0.985849056 |
| Gata3 | Gm38708 | 0.9858386 |
| Ebf3 | Gm38708 | 0.985773461 |
| Pappa2 | C030004G16Rik | 0.985717155 |
| Lhx1 | Gm27151 | 0.985698836 |
| Epha8 | Gm20554 | 0.985547052 |
| Barhl1 | Gm38708 | 0.985457524 |
| Tcf7l2 | Gm38678 | 0.985405288 |
| Bok | C030004G16Rik | 0.985353184 |
| Baiap3 | 4930412O13Rik | 0.985343744 |
| Slc17a6 | Gm39507 | 0.98523278 |
| Arhgap36 | Rmst | 0.985229516 |
| Calb2 | Gm38708 | 0.985187277 |
| Tmem255a | Gm38708 | 0.985138802 |
| Pou4f1 | Gm27151 | 0.985051426 |
| Epha8 | Gm13112 | 0.98501939 |
| Tmem255a | Gm33649 | 0.984940145 |
| Magel2 | 4930412O13Rik | 0.984870409 |
| Igsf1 | Gm38678 | 0.984859624 |
| Fibcd1 | Gm34294 | 0.984799918 |
| Samd14 | Gm2516 | 0.984747894 |
| Tshz2 | Gm39997 | 0.984660678 |
| Irx1 | Gm35395 | 0.984656056 |
| Barhl1 | Gm27151 | 0.984519836 |
| Foxb1 | Gm38580 | 0.984383388 |
| Tmem255a | Gm2516 | 0.98436055 |
| Lhx1 | Gm33649 | 0.984196224 |
| Ctxn3 | Gm27151 | 0.984085397 |
| Lhx1 | Gm38708 | 0.984075184 |
| Pou4f1 | Gm38678 | 0.984025847 |
| Pou4f2 | Gm27151 | 0.984014566 |
| Arhgap36 | Gm13112 | 0.983957298 |
| Medag | C730002L08Rik | 0.983860328 |
| Nkd2 | Gm44781 | 0.983851369 |
| Slc6a12 | Gm38678 | 0.983844927 |
| Pax3 | Gm27151 | 0.983689127 |
| Magel2 | Gm38678 | 0.983647644 |
| Samd14 | Gm33649 | 0.983556744 |
| Pou4f1 | Gm38708 | 0.983503969 |
| Foxb1 | Lhx1os | 0.983440329 |
| Cdhr1 | Gm13112 | 0.983433084 |
| Ctxn3 | Gm38678 | 0.983303036 |
| Arhgap36 | Gm38708 | 0.983255134 |
| Gpr161 | Gm45917 | 0.983237558 |
| Calb2 | Gm27151 | 0.98322454 |
| Pou4f2 | Gm38708 | 0.983136965 |
| Calb2 | Gm33649 | 0.983122506 |
| Barhl1 | Gm33649 | 0.983116836 |
| Magel2 | Gm2516 | 0.983006438 |
| AW551984 | Rmst | 0.982935567 |
| Tcf7l2 | Gm39507 | 0.98292154 |
| Pou4f1 | Gm33649 | 0.982909685 |
| Smyd1 | Rmst | 0.982857815 |
| Magel2 | Gm38708 | 0.98279339 |
| Dgkk | Gm13112 | 0.982789184 |
| Epn3 | Gm38580 | 0.982751638 |
| Tshz2 | Gm45917 | 0.982675078 |
| Ctxn3 | Gm13112 | 0.982668286 |
| Barhl1 | Gm38678 | 0.982587135 |
| Pax3 | Gm38708 | 0.98251492 |
| Trpm3 | Lhx1os | 0.982423527 |
| Pax7 | Gm38678 | 0.982375949 |
| Samd14 | Gm20554 | 0.982371222 |
| Pou4f2 | Gm33649 | 0.982315352 |
| Nkd2 | Gm39507 | 0.982278027 |
| Lhx1 | Gm38678 | 0.9822445 |
| Gpr161 | Gm34344 | 0.982217768 |
| Foxb1 | Gm20554 | 0.982186663 |
| Igsf1 | Rmst | 0.982170586 |
| Cdh23 | Gm35102 | 0.981912689 |
| Shisa6 | Gm36088 | 0.981888745 |
| Pax3 | Gm33649 | 0.981874256 |
| Ntf3 | Gm39178 | 0.98181759 |
| Vipr2 | Gm45917 | 0.981525156 |
| Gata3 | Lhx1os | 0.981508088 |
| Ebf3 | Gm35395 | 0.981502242 |
| Tshz2 | Gm45945 | 0.981229646 |
| Acot5 | Gm41658 | 0.981223687 |
| Pax3 | Gm38678 | 0.981073139 |
| Dgkk | Gm38708 | 0.980867568 |
| Foxb1 | Gm38708 | 0.980834854 |
| Shox2 | Gm27151 | 0.980834014 |
| Tcf7l2 | Gm35395 | 0.980824292 |
| Plpp4 | Gm44781 | 0.980822692 |
| Fibcd1 | Gm45917 | 0.980784564 |
| Fibcd1 | Gm29906 | 0.980631355 |
| Pou4f2 | Gm38678 | 0.98050879 |
| 2210418O10Rik | Gm39997 | 0.98044938 |
| Irx1 | Gm38678 | 0.980426985 |
| Epha8 | Gm39507 | 0.980247805 |
| Epha8 | Gm33649 | 0.980169481 |
| Dgkk | 4930412O13Rik | 0.979892909 |
| Cdhr1 | Gm39507 | 0.979882956 |
| Gdpd2 | Gm39507 | 0.979801698 |
| Arhgap36 | Gm33649 | 0.979685446 |
| Tmem255a | Gm27151 | 0.979446772 |
| Trpm3 | Gm39507 | 0.9792801 |
| Epha8 | Gm27151 | 0.979271434 |
| Medag | C030004G16Rik | 0.979267642 |
| Adamts19 | Gm44781 | 0.979130397 |
| Samd14 | Gm38708 | 0.97905326 |
| Dsc3 | Gm41322 | 0.978868836 |
| Slc6a12 | 4930412O13Rik | 0.978783743 |
| Sfrp5 | Gm35395 | 0.97862902 |
| Tenm3 | Gm42071 | 0.978556689 |
| Syne4 | Gm39507 | 0.978542624 |
| Tmem255a | Gm13112 | 0.978499155 |
| Shox2 | Gm33649 | 0.978416341 |
| Epn3 | Gm39507 | 0.978393322 |
| Arhgap36 | Gm27151 | 0.978354378 |
| Adcy8 | Gm39178 | 0.978260075 |
| Samd14 | Gm27151 | 0.97814757 |
| Shox2 | 4930412O13Rik | 0.978128267 |
| Ctxn3 | Rmst | 0.978073098 |
| Irx1 | Gm39507 | 0.977912009 |
| Krt80 | 1700121C08Rik | 0.977808186 |
| Tenm2 | Gm42071 | 0.977700645 |
| Slc17a6 | Gm38708 | 0.97764174 |
| Tmem40 | C030004G16Rik | 0.977607764 |
| Magel2 | Gm38580 | 0.977589231 |
| Cabp7 | Gm36088 | 0.977477768 |
| Arhgap36 | 4930412O13Rik | 0.977381987 |
| Pappa2 | Gm32412 | 0.977337844 |
| AW551984 | Gm13112 | 0.977321283 |
| Calb2 | Rmst | 0.977315249 |
| Cdhr1 | 4930412O13Rik | 0.976829544 |
| Agt | Rmst | 0.976686 |
| Sox14 | Gm36088 | 0.97667963 |
| Shisa6 | Gm45917 | 0.976672207 |
| Vwc2l | Gm42071 | 0.976550841 |
| Igsf1 | Gm39507 | 0.97654157 |
| Ctxn3 | Gm20554 | 0.976487507 |
| Fibcd1 | Gm35102 | 0.976291361 |
| Dgkk | Gm27151 | 0.976241677 |
| Calb2 | Gm38678 | 0.976159268 |
| Dgkk | Gm33649 | 0.976073611 |
| Prlhr | Gm20554 | 0.975970644 |
| Ntng1 | Gm39178 | 0.975953871 |
| Sox14 | Gm44781 | 0.975897509 |
| Baiap3 | Gm38678 | 0.975866778 |
| Slc6a12 | Gm38708 | 0.975866406 |
| Epha8 | Gm38580 | 0.975297395 |
| Trpm3 | Rmst | 0.975006016 |
| Ndst4 | Gm38678 | 0.974941952 |
| Smyd1 | Gm2516 | 0.974869543 |
| Adcy8 | Gm29906 | 0.974806069 |
| Cbln3 | Gm20554 | 0.974756313 |
| Slc6a12 | Gm2516 | 0.974461926 |
| Baiap3 | Gm39507 | 0.974352696 |
| Dgkk | Rmst | 0.974181999 |
| AW551984 | Gm38708 | 0.97416235 |
| Foxb1 | Gm35395 | 0.974055123 |
| Cdh23 | Gm44781 | 0.974047814 |
| Epn3 | Lhx1os | 0.973599784 |
| AW551984 | Gm2516 | 0.973452094 |
| Arhgap36 | Gm2516 | 0.973441796 |
| Shox2 | Gm13112 | 0.973380515 |
| Pirt | Gm34344 | 0.973230436 |
| Irx1 | Gm2516 | 0.973181057 |
| Shox2 | Gm44781 | 0.97304091 |
| Samd14 | Gm13112 | 0.972909548 |
| Epha8 | Gm38678 | 0.97268444 |
| Tcf7l2 | Gm2516 | 0.972658876 |
| Prlhr | Gm38708 | 0.97261832 |
| Cbln3 | 4930412O13Rik | 0.972513125 |
| Cbln3 | Gm13112 | 0.972441931 |
| Trpm3 | Gm35395 | 0.972412031 |
| Htr4 | Gm36316 | 0.972329186 |
| Lhx9 | Rmst | 0.972127103 |
| Sox14 | Gm38678 | 0.97209638 |
| Baiap3 | Gm2516 | 0.972037168 |
| AW551984 | Gm33649 | 0.971852619 |
| Tmem255a | Gm38580 | 0.971815942 |
| 2210418O10Rik | Gm34294 | 0.971804855 |
| Cdhr1 | Gm20554 | 0.971682561 |
| Gpr161 | Gm45945 | 0.971646413 |
| Fibcd1 | Gm42071 | 0.971418668 |
| Pax7 | 4930412O13Rik | 0.971291057 |
| Fibcd1 | Gm39997 | 0.971219157 |
| Lmo1 | Gm33649 | 0.971182531 |
| Gata3 | Gm35395 | 0.971141384 |
| Lmo1 | Gm27151 | 0.971102346 |
| Smyd1 | Gm33649 | 0.97101797 |
| Nkd2 | Gm33649 | 0.971000142 |
| AW551984 | Gm27151 | 0.97088688 |
| Agt | Gm39178 | 0.970698967 |
| Nkd2 | Gm39178 | 0.970678936 |
| Slc17a6 | Gm38580 | 0.970361649 |
| Tmem40 | Gm32412 | 0.97033824 |
| Ctxn3 | 4930412O13Rik | 0.970294621 |
| Gm29779 | Gm39507 | 0.970250565 |
| AW551984 | 4930412O13Rik | 0.969965553 |
| Pax7 | Gm27151 | 0.969961618 |
| Gata3 | Gm39507 | 0.969901864 |
| Slc17a6 | Rmst | 0.969891378 |
| Sfrp5 | Gm39507 | 0.96982892 |
| Epha8 | 4930412O13Rik | 0.969806636 |
| Lmo1 | Gm38678 | 0.969757681 |
| Agt | Gm33649 | 0.969736561 |
| Arhgap36 | Gm39507 | 0.969687855 |
| Lhx9 | Gm39507 | 0.969473486 |
| Tshz2 | Gm29906 | 0.969194045 |
| Smyd1 | Gm20554 | 0.969150233 |
| Nkd2 | Gm27151 | 0.969139558 |
| Agt | Gm2516 | 0.968643569 |
| Slc6a12 | Gm38580 | 0.968526054 |
| Cbln3 | Gm38708 | 0.968495584 |
| Lhx5 | Rmst | 0.968336564 |
| Nnat | Gm2516 | 0.968266372 |
| Pirt | Gm34294 | 0.968251634 |
| Cabp7 | Gm45917 | 0.968138791 |
| Shisa6 | Gm34344 | 0.968054218 |
| Lhx1 | Rmst | 0.967663884 |
| Gpr161 | Gm34294 | 0.967504951 |
| Vipr2 | Gm36088 | 0.967481016 |
| Gm29779 | Gm33649 | 0.967368931 |
| Acot5 | Gm38615 | 0.967211949 |
| Igsf1 | Gm2516 | 0.967198218 |
| Prlhr | Gm38580 | 0.967183216 |
| Sfrp5 | Rmst | 0.967061502 |
| Vipr2 | Gm34294 | 0.966976508 |
| Ndst4 | Gm36088 | 0.966936353 |
| Agt | Gm27151 | 0.966901696 |
| Tmem255a | Gm38678 | 0.966845944 |
| Gm29779 | Gm27151 | 0.966755892 |
| Irx2 | Rmst | 0.966681348 |
| Pax7 | Gm33649 | 0.966185332 |
| Cabp7 | Gm34294 | 0.966157303 |
| Cdhr1 | Gm44781 | 0.966135904 |
| Epn3 | Rmst | 0.966049594 |
| Agt | Gm20554 | 0.965979449 |
| Dgkk | Gm38678 | 0.965880727 |
| Arhgap36 | Gm38678 | 0.965790427 |
| Tmem255a | 4930412O13Rik | 0.965787505 |
| Ctxn3 | Gm38708 | 0.965504771 |
| Gpr161 | Gm36088 | 0.965501899 |
| Cdh23 | Gm34294 | 0.965448307 |
| Npffr1 | Gm45945 | 0.965440926 |
| Pou4f2 | Rmst | 0.964941866 |
| Bok | Gm40055 | 0.964761435 |
| Samd14 | Gm38678 | 0.964728091 |
| Smyd1 | Gm38708 | 0.964703809 |
| Irx2 | Gm39507 | 0.964505038 |
| Cdh23 | Gm29906 | 0.964228107 |
| Calb2 | Gm39507 | 0.963994971 |
| Prlhr | Gm39507 | 0.963882985 |
| Plpp4 | Gm39507 | 0.963817979 |
| Smyd1 | Gm27151 | 0.963717619 |
| Cabp7 | Gm44781 | 0.96365648 |
| Ebf3 | Gm39507 | 0.963639031 |
| Pax3 | Rmst | 0.963309425 |
| Barhl1 | Rmst | 0.963076613 |
| Pax7 | Gm13112 | 0.963069013 |
| Cdhr1 | Rmst | 0.962794402 |
| Tmem40 | C730002L08Rik | 0.962778714 |
| Gata3 | Rmst | 0.962713101 |
| AW551984 | Gm39507 | 0.962681022 |
| Pappa2 | Gm40055 | 0.962588834 |
| Lmo1 | 4930412O13Rik | 0.962543766 |
| Lhx5 | Gm39507 | 0.962367474 |
| Ebf3 | Rmst | 0.962208046 |
| Epn3 | Gm35395 | 0.962159578 |
| Cbln3 | Gm27151 | 0.962132505 |
| Ndst4 | Gm27151 | 0.962080904 |
| Fibcd1 | Gm45945 | 0.961991735 |
| Lmo1 | Gm39507 | 0.961836341 |
| Bok | Gm32412 | 0.961711914 |
| Slc17a6 | Gm44781 | 0.961535156 |
| Samd14 | 4930412O13Rik | 0.961496477 |
| Epha8 | Lhx1os | 0.96146346 |
| Pax7 | Gm44781 | 0.961383951 |
| Agt | Gm13112 | 0.961327316 |
| Ctxn3 | Gm44781 | 0.961243306 |
| Gdpd2 | Gm39178 | 0.961188758 |
| Syne4 | Gm2516 | 0.961017649 |
| Nnat | Gm39178 | 0.960992639 |
| Dgkk | Gm2516 | 0.960802966 |
| Nkd2 | Gm38678 | 0.960590856 |
| Prlhr | Lhx1os | 0.960566643 |
| Epn3 | Gm2516 | 0.96047016 |
| Cbln3 | Gm33649 | 0.960457251 |
| Magel2 | Lhx1os | 0.960425529 |
| Sox14 | Gm27151 | 0.960301183 |
| Epha8 | Gm2516 | 0.960142697 |
| Htr4 | Gm34344 | 0.960034968 |
| Tmem40 | Gm29906 | -0.960675257 |
| Pappa2 | Gm42071 | -0.961555239 |
| Tmem40 | Gm35102 | -0.961608785 |
| 2210418O10Rik | Gm41658 | -0.962638337 |
| Tmem40 | Gm42071 | -0.963949721 |
| Tenm3 | Gm40055 | -0.964110309 |
| Npffr1 | Gm32442 | -0.964433051 |
| Adamtsl5 | Gm33585 | -0.965108137 |
| Tenm3 | Gm32442 | -0.968907855 |
| Pappa2 | Gm35102 | -0.969423386 |
| Pappa2 | Gm34294 | -0.971215183 |
| Adcy8 | Gm41658 | -0.971402926 |
| 2210418O10Rik | C030004G16Rik | -0.972545995 |
| Adcy8 | Gm40055 | -0.975250914 |
| Adcy8 | C030004G16Rik | -0.977308151 |
| Pappa2 | Gm39997 | -0.979173235 |
| Htr2a | Gm29906 | -0.980911784 |
| Htr2a | Gm34294 | -0.985077595 |
| Gm14296 | Gm42071 | -0.985702346 |
| 2210418O10Rik | Gm40055 | -0.988744254 |
| Htr2a | Gm35102 | -0.989585592 |
| Pappa2 | Gm29906 | -0.98977281 |
| Gm14296 | Gm39997 | -0.993884205 |
| Gm14296 | Gm33585 | -0.997169118 |
