# Supplementary material for: Protective effects and regulatory pathways of melatonin in traumatic brain injury mice model: Transcriptomics and bioinformatics analysis
Source: Front Mol Neurosci. 2022 Sep 9;15:974060. doi: 10.3389/fnmol.2022.974060 (PMC9500234; doi:10.3389/fnmol.2022.974060)
Supplement: Supplementary file 3 [file Table_3.DOCX]

| Supplemental Table 3. *Cis*-regulation of DEmRNAs and DElncRNAs | | | |
| --- | --- | --- | --- |
| mRNA | mRNA chromosome | lncRNA | lncRNA chromosome |
| Gata3 | NC_000068.7 | 4930412O13Rik | NC_000068.7 |
| Trpm3 | NC_000085.6 | Gm27151 | NC_000085.6 |
| Pirt | NC_000077.6 | Gm45945 | NC_000077.6 |
| Irx1 | NC_000079.6 | Gm20554 | NC_000079.6 |
| Lhx1 | NC_000077.6 | Lhx1os | NC_000077.6 |
| Irx1 | NC_000079.6 | Gm38580 | NC_000079.6 |
| Trpm3 | NC_000085.6 | Gm33649 | NC_000085.6 |
| Sfrp5 | NC_000085.6 | Gm27151 | NC_000085.6 |
| Tmem255a | NC_000086.7 | Gm39507 | NC_000086.7 |
| Irx2 | NC_000079.6 | Gm38580 | NC_000079.6 |
| Sfrp5 | NC_000085.6 | Gm33649 | NC_000085.6 |
| Tcf7l2 | NC_000085.6 | Gm27151 | NC_000085.6 |
| Prlhr | NC_000085.6 | Gm27151 | NC_000085.6 |
| Pirt | NC_000077.6 | Gm45917 | NC_000077.6 |
| Irx2 | NC_000079.6 | Gm20554 | NC_000079.6 |
| Lhx5 | NC_000071.6 | Gm35395 | NC_000071.6 |
| Prlhr | NC_000085.6 | Gm33649 | NC_000085.6 |
| Lmo1 | NC_000073.6 | Gm44781 | NC_000073.6 |
| Tshz2 | NC_000068.7 | Gm34294 | NC_000068.7 |
| Epha8 | NC_000070.6 | Gm13112 | NC_000070.6 |
| Fibcd1 | NC_000068.7 | Gm34294 | NC_000068.7 |
| Tshz2 | NC_000068.7 | Gm39997 | NC_000068.7 |
| Plpp4 | NC_000073.6 | Gm44781 | NC_000073.6 |
| 2210418O10Rik | NC_000068.7 | Gm39997 | NC_000068.7 |
| Gdpd2 | NC_000086.7 | Gm39507 | NC_000086.7 |
| Tenm2 | NC_000077.6 | Gm42071 | NC_000077.6 |
| Sox14 | NC_000075.6 | Gm36088 | NC_000075.6 |
| Shisa6 | NC_000077.6 | Gm45917 | NC_000077.6 |
| Igsf1 | NC_000086.7 | Gm39507 | NC_000086.7 |
| Slc6a12 | NC_000072.6 | Gm38708 | NC_000072.6 |
| Epn3 | NC_000077.6 | Lhx1os | NC_000077.6 |
| Htr4 | NC_000084.6 | Gm36316 | NC_000084.6 |
| Sox14 | NC_000075.6 | Gm38678 | NC_000075.6 |
| 2210418O10Rik | NC_000068.7 | Gm34294 | NC_000068.7 |
| Fibcd1 | NC_000068.7 | Gm39997 | NC_000068.7 |
| Agt | NC_000074.6 | Gm39178 | NC_000074.6 |
| Npffr1 | NC_000076.6 | Gm45945 | NC_000077.6 |
| Pax7 | NC_000070.6 | Gm13112 | NC_000070.6 |
| Slc17a6 | NC_000073.6 | Gm44781 | NC_000073.6 |
| Pappa2 | NC_000067.6 | Gm29906 | NC_000067.6 |
| Gm14296 | NC_000068.7 | Gm39997 | NC_000068.7 |
