# Supplementary material for: Protective effects and regulatory pathways of melatonin in traumatic brain injury mice model: Transcriptomics and bioinformatics analysis
Source: Front Mol Neurosci. 2022 Sep 9;15:974060. doi: 10.3389/fnmol.2022.974060 (PMC9500234; doi:10.3389/fnmol.2022.974060)
Supplement: Supplementary file 4 [file Table_4.DOCX]

| Supplemental Table 4. *Trans*-regulation of DEmRNAs and DElncRNAs | | | |
| --- | --- | --- | --- |
| mRNA | mRNA chromosome | lncRNA | lncRNA chromosome |
| Cbln3 | NC_000080.6 | Gm35395 | NC_000071.6 |
| Calb2 | NC_000074.6 | Gm38580 | NC_000079.6 |
| Sfrp5 | NC_000085.6 | 4930412O13Rik | NC_000068.7 |
| Ebf3 | NC_000073.6 | 4930412O13Rik | NC_000068.7 |
| Tcf7l2 | NC_000085.6 | Gm20554 | NC_000079.6 |
| Trpm3 | NC_000085.6 | Gm13112 | NC_000070.6 |
| Dgkk | NC_000086.7 | Gm38580 | NC_000079.6 |
| Cbln3 | NC_000080.6 | Lhx1os | NC_000077.6 |
| Trpm3 | NC_000085.6 | 4930412O13Rik | NC_000068.7 |
| Baiap3 | NC_000083.6 | Gm38580 | NC_000079.6 |
| Pou4f2 | NC_000074.6 | Lhx1os | NC_000077.6 |
| Pax3 | NC_000067.6 | Lhx1os | NC_000077.6 |
| Lhx1 | NC_000077.6 | Gm38580 | NC_000079.6 |
| Igsf1 | NC_000086.7 | Gm38580 | NC_000079.6 |
| Pou4f2 | NC_000074.6 | Gm38580 | NC_000079.6 |
| Foxb1 | NC_000075.6 | 4930412O13Rik | NC_000068.7 |
| Samd14 | NC_000077.6 | Gm39507 | NC_000086.7 |
| Barhl1 | NC_000068.7 | Lhx1os | NC_000077.6 |
| Prlhr | NC_000085.6 | Gm38678 | NC_000075.6 |
| Igsf1 | NC_000086.7 | Gm20554 | NC_000079.6 |
| Epn3 | NC_000077.6 | Gm27151 | NC_000085.6 |
| Pax3 | NC_000067.6 | Gm38580 | NC_000079.6 |
| Slc6a12 | NC_000072.6 | Gm39507 | NC_000086.7 |
| Irx2 | NC_000079.6 | 4930412O13Rik | NC_000068.7 |
| Slc17a6 | NC_000073.6 | Gm27151 | NC_000085.6 |
| Sfrp5 | NC_000085.6 | Gm13112 | NC_000070.6 |
| Htr4 | NC_000084.6 | Gm45945 | NC_000077.6 |
| Lhx5 | NC_000071.6 | Gm38580 | NC_000079.6 |
| Calb2 | NC_000074.6 | Lhx1os | NC_000077.6 |
| Lhx9 | NC_000067.6 | 4930412O13Rik | NC_000068.7 |
| Arhgap36 | NC_000086.7 | Gm38580 | NC_000079.6 |
| Gata3 | NC_000068.7 | Gm27151 | NC_000085.6 |
| Gata3 | NC_000068.7 | Gm38678 | NC_000075.6 |
| Barhl1 | NC_000068.7 | Gm38580 | NC_000079.6 |
| Pou4f1 | NC_000080.6 | Lhx1os | NC_000077.6 |
| Epn3 | NC_000077.6 | 4930412O13Rik | NC_000068.7 |
| Baiap3 | NC_000083.6 | Gm20554 | NC_000079.6 |
| Gata3 | NC_000068.7 | Gm13112 | NC_000070.6 |
| Lhx9 | NC_000067.6 | Gm13112 | NC_000070.6 |
| Epn3 | NC_000077.6 | Gm33649 | NC_000085.6 |
| Slc17a6 | NC_000073.6 | Gm33649 | NC_000085.6 |
| Dgkk | NC_000086.7 | Lhx1os | NC_000077.6 |
| Lhx9 | NC_000067.6 | Gm38580 | NC_000079.6 |
| Tcf7l2 | NC_000085.6 | Gm38580 | NC_000079.6 |
| Irx2 | NC_000079.6 | Gm13112 | NC_000070.6 |
| Lhx5 | NC_000071.6 | Lhx1os | NC_000077.6 |
| Bok | NC_000067.6 | C730002L08Rik | NC_000085.6 |
| Ebf3 | NC_000073.6 | Gm13112 | NC_000070.6 |
| Magel2 | NC_000073.6 | Gm33649 | NC_000085.6 |
| Tenm2 | NC_000077.6 | Gm33585 | NC_000077.6 |
| Foxb1 | NC_000075.6 | Gm38678 | NC_000075.6 |
| Tcf7l2 | NC_000085.6 | Gm13112 | NC_000070.6 |
| Pou4f1 | NC_000080.6 | Gm38580 | NC_000079.6 |
| Trpm3 | NC_000085.6 | Gm20554 | NC_000079.6 |
| Trpm3 | NC_000085.6 | Gm38678 | NC_000075.6 |
| Igsf1 | NC_000086.7 | Gm13112 | NC_000070.6 |
| Gata3 | NC_000068.7 | Gm33649 | NC_000085.6 |
| Dgkk | NC_000086.7 | Gm35395 | NC_000071.6 |
| Pou4f2 | NC_000074.6 | Gm35395 | NC_000071.6 |
| Lhx5 | NC_000071.6 | 4930412O13Rik | NC_000068.7 |
| Slc17a6 | NC_000073.6 | Gm38678 | NC_000075.6 |
| Pax3 | NC_000067.6 | Gm35395 | NC_000071.6 |
| Magel2 | NC_000073.6 | Gm20554 | NC_000079.6 |
| Magel2 | NC_000073.6 | Gm39507 | NC_000086.7 |
| Stac | NC_000075.6 | Gm34344 | NC_000068.7 |
| Sfrp5 | NC_000085.6 | Gm38678 | NC_000075.6 |
| Prlhr | NC_000085.6 | 4930412O13Rik | NC_000068.7 |
| Smyd1 | NC_000072.6 | Gm39507 | NC_000086.7 |
| AW551984 | NC_000075.6 | Gm38580 | NC_000079.6 |
| Lhx5 | NC_000071.6 | Gm13112 | NC_000070.6 |
| Magel2 | NC_000073.6 | Gm27151 | NC_000085.6 |
| Tcf7l2 | NC_000085.6 | Gm33649 | NC_000085.6 |
| 2210418O10Rik | NC_000068.7 | Gm29906 | NC_000067.6 |
| Lhx9 | NC_000067.6 | Gm20554 | NC_000079.6 |
| Lhx1 | NC_000077.6 | Gm35395 | NC_000071.6 |
| Calb2 | NC_000074.6 | Gm35395 | NC_000071.6 |
| Lhx9 | NC_000067.6 | Gm27151 | NC_000085.6 |
| Ebf3 | NC_000073.6 | Gm27151 | NC_000085.6 |
| Barhl1 | NC_000068.7 | Gm35395 | NC_000071.6 |
| Arhgap36 | NC_000086.7 | Gm20554 | NC_000079.6 |
| Cbln3 | NC_000080.6 | Gm38580 | NC_000079.6 |
| Epn3 | NC_000077.6 | Gm13112 | NC_000070.6 |
| Slc6a12 | NC_000072.6 | Gm33649 | NC_000085.6 |
| Foxb1 | NC_000075.6 | Gm13112 | NC_000070.6 |
| Irx1 | NC_000079.6 | Gm13112 | NC_000070.6 |
| Ebf3 | NC_000073.6 | Gm38678 | NC_000075.6 |
| Tcf7l2 | NC_000085.6 | Gm38708 | NC_000072.6 |
| Slc17a6 | NC_000073.6 | Gm13112 | NC_000070.6 |
| Pou4f1 | NC_000080.6 | 4930412O13Rik | NC_000068.7 |
| Krt80 | NC_000081.6 | Gm30848 | NC_000068.7 |
| Epn3 | NC_000077.6 | Gm38678 | NC_000075.6 |
| Tmem255a | NC_000086.7 | Rmst | NC_000076.6 |
| Calb2 | NC_000074.6 | Gm20554 | NC_000079.6 |
| Irx2 | NC_000079.6 | Lhx1os | NC_000077.6 |
| Lhx9 | NC_000067.6 | Lhx1os | NC_000077.6 |
| Egfem1 | NC_000069.6 | Gm33585 | NC_000077.6 |
| Irx2 | NC_000079.6 | Gm27151 | NC_000085.6 |
| Lhx9 | NC_000067.6 | Gm33649 | NC_000085.6 |
| Pou4f1 | NC_000080.6 | Gm35395 | NC_000071.6 |
| Igsf1 | NC_000086.7 | 4930412O13Rik | NC_000068.7 |
| Igsf1 | NC_000086.7 | Lhx1os | NC_000077.6 |
| Igsf1 | NC_000086.7 | Gm33649 | NC_000085.6 |
| Syne4 | NC_000073.6 | Gm39178 | NC_000074.6 |
| Sfrp5 | NC_000085.6 | Gm20554 | NC_000079.6 |
| Baiap3 | NC_000083.6 | Lhx1os | NC_000077.6 |
| Tenm2 | NC_000077.6 | Gm39997 | NC_000068.7 |
| Igsf1 | NC_000086.7 | Gm27151 | NC_000085.6 |
| Olfr78 | NC_000073.6 | Gm30848 | NC_000068.7 |
| Foxb1 | NC_000075.6 | Gm27151 | NC_000085.6 |
| Mdga1 | NC_000083.6 | Gm41322 | NC_000081.6 |
| Ebf3 | NC_000073.6 | Gm38580 | NC_000079.6 |
| Slc17a6 | NC_000073.6 | 4930412O13Rik | NC_000068.7 |
| Lhx1 | NC_000077.6 | 4930412O13Rik | NC_000068.7 |
| 2210418O10Rik | NC_000068.7 | Gm42071 | NC_000077.6 |
| Sfrp5 | NC_000085.6 | Gm38580 | NC_000079.6 |
| Ebf3 | NC_000073.6 | Gm33649 | NC_000085.6 |
| Irx2 | NC_000079.6 | Gm38678 | NC_000075.6 |
| Tmem40 | NC_000072.6 | Gm40055 | NC_000069.6 |
| Igsf1 | NC_000086.7 | Gm38708 | NC_000072.6 |
| Lhx1 | NC_000077.6 | Gm13112 | NC_000070.6 |
| Barhl1 | NC_000068.7 | 4930412O13Rik | NC_000068.7 |
| Slc6a12 | NC_000072.6 | Gm27151 | NC_000085.6 |
| Lhx5 | NC_000071.6 | Gm20554 | NC_000079.6 |
| Slc27a2 | NC_000068.7 | Gm39178 | NC_000074.6 |
| Tcf7l2 | NC_000085.6 | 4930412O13Rik | NC_000068.7 |
| Epn3 | NC_000077.6 | Gm38708 | NC_000072.6 |
| Epn3 | NC_000077.6 | Gm20554 | NC_000079.6 |
| Ctxn3 | NC_000084.6 | Gm39507 | NC_000086.7 |
| Epha8 | NC_000070.6 | Rmst | NC_000076.6 |
| Pax3 | NC_000067.6 | 4930412O13Rik | NC_000068.7 |
| Irx2 | NC_000079.6 | Gm33649 | NC_000085.6 |
| Pou4f2 | NC_000074.6 | 4930412O13Rik | NC_000068.7 |
| Baiap3 | NC_000083.6 | Gm13112 | NC_000070.6 |
| Arhgap36 | NC_000086.7 | Lhx1os | NC_000077.6 |
| Magel2 | NC_000073.6 | Gm13112 | NC_000070.6 |
| Lhx9 | NC_000067.6 | Gm38678 | NC_000075.6 |
| Barhl1 | NC_000068.7 | Gm13112 | NC_000070.6 |
| Prlhr | NC_000085.6 | Gm13112 | NC_000070.6 |
| Pou4f1 | NC_000080.6 | Gm13112 | NC_000070.6 |
| Irx1 | NC_000079.6 | Lhx1os | NC_000077.6 |
| Irx1 | NC_000079.6 | Gm33649 | NC_000085.6 |
| Trpm3 | NC_000085.6 | Gm38580 | NC_000079.6 |
| Lhx1 | NC_000077.6 | Gm20554 | NC_000079.6 |
| Pou4f2 | NC_000074.6 | Gm13112 | NC_000070.6 |
| Irx1 | NC_000079.6 | Gm27151 | NC_000085.6 |
| Plpp4 | NC_000073.6 | Gm39178 | NC_000074.6 |
| Dgkk | NC_000086.7 | Gm20554 | NC_000079.6 |
| Pax3 | NC_000067.6 | Gm13112 | NC_000070.6 |
| Lhx5 | NC_000071.6 | Gm27151 | NC_000085.6 |
| AW551984 | NC_000075.6 | Gm20554 | NC_000079.6 |
| Calb2 | NC_000074.6 | Gm13112 | NC_000070.6 |
| Slc6a12 | NC_000072.6 | Gm20554 | NC_000079.6 |
| Ebf3 | NC_000073.6 | Lhx1os | NC_000077.6 |
| Ebf3 | NC_000073.6 | Gm20554 | NC_000079.6 |
| Olfr78 | NC_000073.6 | 1700121C08Rik | NC_000070.6 |
| Gata3 | NC_000068.7 | Gm20554 | NC_000079.6 |
| Foxb1 | NC_000075.6 | Gm33649 | NC_000085.6 |
| Cdhr1 | NC_000080.6 | Gm38678 | NC_000075.6 |
| Irx1 | NC_000079.6 | 4930412O13Rik | NC_000068.7 |
| Adcy8 | NC_000081.6 | Gm35102 | NC_000077.6 |
| Slc6a12 | NC_000072.6 | Gm13112 | NC_000070.6 |
| Lhx9 | NC_000067.6 | Gm38708 | NC_000072.6 |
| Pou4f2 | NC_000074.6 | Gm20554 | NC_000079.6 |
| Tmem255a | NC_000086.7 | Gm20554 | NC_000079.6 |
| Gm29779 | NC_000071.6 | Gm44781 | NC_000073.6 |
| Lhx5 | NC_000071.6 | Gm33649 | NC_000085.6 |
| Shox2 | NC_000069.6 | Gm38678 | NC_000075.6 |
| Cartpt | NC_000079.6 | Gm39178 | NC_000074.6 |
| Slc6a12 | NC_000072.6 | Rmst | NC_000076.6 |
| Agt | NC_000074.6 | Gm39507 | NC_000086.7 |
| AW551984 | NC_000075.6 | Lhx1os | NC_000077.6 |
| Arhgap36 | NC_000086.7 | Gm35395 | NC_000071.6 |
| Trpm3 | NC_000085.6 | Gm38708 | NC_000072.6 |
| Tcf7l2 | NC_000085.6 | Lhx1os | NC_000077.6 |
| Baiap3 | NC_000083.6 | Gm35395 | NC_000071.6 |
| Pax3 | NC_000067.6 | Gm20554 | NC_000079.6 |
| Barhl1 | NC_000068.7 | Gm20554 | NC_000079.6 |
| Sfrp5 | NC_000085.6 | Gm38708 | NC_000072.6 |
| Sfrp5 | NC_000085.6 | Lhx1os | NC_000077.6 |
| 2210418O10Rik | NC_000068.7 | Gm35102 | NC_000077.6 |
| Lhx5 | NC_000071.6 | Gm38678 | NC_000075.6 |
| Adamts19 | NC_000084.6 | Gm36088 | NC_000075.6 |
| Slc17a6 | NC_000073.6 | Gm20554 | NC_000079.6 |
| Magel2 | NC_000073.6 | Rmst | NC_000076.6 |
| Ctxn3 | NC_000084.6 | Gm33649 | NC_000085.6 |
| Htr4 | NC_000084.6 | Gm45917 | NC_000077.6 |
| Irx1 | NC_000079.6 | Rmst | NC_000076.6 |
| Baiap3 | NC_000083.6 | Gm33649 | NC_000085.6 |
| AW551984 | NC_000075.6 | Gm35395 | NC_000071.6 |
| Irx2 | NC_000079.6 | Gm38708 | NC_000072.6 |
| Cdhr1 | NC_000080.6 | Gm27151 | NC_000085.6 |
| Baiap3 | NC_000083.6 | Gm38708 | NC_000072.6 |
| Gata3 | NC_000068.7 | Gm38580 | NC_000079.6 |
| Irx1 | NC_000079.6 | Gm38708 | NC_000072.6 |
| Igsf1 | NC_000086.7 | Gm35395 | NC_000071.6 |
| Calb2 | NC_000074.6 | 4930412O13Rik | NC_000068.7 |
| Tcf7l2 | NC_000085.6 | Rmst | NC_000076.6 |
| Cdhr1 | NC_000080.6 | Gm33649 | NC_000085.6 |
| Lhx5 | NC_000071.6 | Gm38708 | NC_000072.6 |
| C1ql2 | NC_000067.6 | Gm34344 | NC_000068.7 |
| Npffr1 | NC_000076.6 | Gm45917 | NC_000077.6 |
| Baiap3 | NC_000083.6 | Gm27151 | NC_000085.6 |
| Samd14 | NC_000077.6 | Rmst | NC_000076.6 |
| Lhx9 | NC_000067.6 | Gm35395 | NC_000071.6 |
| Irx2 | NC_000079.6 | Gm35395 | NC_000071.6 |
| Pou4f1 | NC_000080.6 | Gm20554 | NC_000079.6 |
| Baiap3 | NC_000083.6 | Rmst | NC_000076.6 |
| Gata3 | NC_000068.7 | Gm38708 | NC_000072.6 |
| Ebf3 | NC_000073.6 | Gm38708 | NC_000072.6 |
| Pappa2 | NC_000067.6 | C030004G16Rik | NC_000084.6 |
| Lhx1 | NC_000077.6 | Gm27151 | NC_000085.6 |
| Epha8 | NC_000070.6 | Gm20554 | NC_000079.6 |
| Barhl1 | NC_000068.7 | Gm38708 | NC_000072.6 |
| Tcf7l2 | NC_000085.6 | Gm38678 | NC_000075.6 |
| Bok | NC_000067.6 | C030004G16Rik | NC_000084.6 |
| Baiap3 | NC_000083.6 | 4930412O13Rik | NC_000068.7 |
| Slc17a6 | NC_000073.6 | Gm39507 | NC_000086.7 |
| Arhgap36 | NC_000086.7 | Rmst | NC_000076.6 |
| Calb2 | NC_000074.6 | Gm38708 | NC_000072.6 |
| Tmem255a | NC_000086.7 | Gm38708 | NC_000072.6 |
| Pou4f1 | NC_000080.6 | Gm27151 | NC_000085.6 |
| Tmem255a | NC_000086.7 | Gm33649 | NC_000085.6 |
| Magel2 | NC_000073.6 | 4930412O13Rik | NC_000068.7 |
| Igsf1 | NC_000086.7 | Gm38678 | NC_000075.6 |
| Samd14 | NC_000077.6 | Gm2516 | NC_000074.6 |
| Irx1 | NC_000079.6 | Gm35395 | NC_000071.6 |
| Barhl1 | NC_000068.7 | Gm27151 | NC_000085.6 |
| Foxb1 | NC_000075.6 | Gm38580 | NC_000079.6 |
| Tmem255a | NC_000086.7 | Gm2516 | NC_000074.6 |
| Lhx1 | NC_000077.6 | Gm33649 | NC_000085.6 |
| Ctxn3 | NC_000084.6 | Gm27151 | NC_000085.6 |
| Lhx1 | NC_000077.6 | Gm38708 | NC_000072.6 |
| Pou4f1 | NC_000080.6 | Gm38678 | NC_000075.6 |
| Pou4f2 | NC_000074.6 | Gm27151 | NC_000085.6 |
| Arhgap36 | NC_000086.7 | Gm13112 | NC_000070.6 |
| Medag | NC_000071.6 | C730002L08Rik | NC_000085.6 |
| Nkd2 | NC_000079.6 | Gm44781 | NC_000073.6 |
| Slc6a12 | NC_000072.6 | Gm38678 | NC_000075.6 |
| Pax3 | NC_000067.6 | Gm27151 | NC_000085.6 |
| Magel2 | NC_000073.6 | Gm38678 | NC_000075.6 |
| Samd14 | NC_000077.6 | Gm33649 | NC_000085.6 |
| Pou4f1 | NC_000080.6 | Gm38708 | NC_000072.6 |
| Foxb1 | NC_000075.6 | Lhx1os | NC_000077.6 |
| Cdhr1 | NC_000080.6 | Gm13112 | NC_000070.6 |
| Ctxn3 | NC_000084.6 | Gm38678 | NC_000075.6 |
| Arhgap36 | NC_000086.7 | Gm38708 | NC_000072.6 |
| Gpr161 | NC_000067.6 | Gm45917 | NC_000077.6 |
| Calb2 | NC_000074.6 | Gm27151 | NC_000085.6 |
| Pou4f2 | NC_000074.6 | Gm38708 | NC_000072.6 |
| Calb2 | NC_000074.6 | Gm33649 | NC_000085.6 |
| Barhl1 | NC_000068.7 | Gm33649 | NC_000085.6 |
| Magel2 | NC_000073.6 | Gm2516 | NC_000074.6 |
| AW551984 | NC_000075.6 | Rmst | NC_000076.6 |
| Tcf7l2 | NC_000085.6 | Gm39507 | NC_000086.7 |
| Pou4f1 | NC_000080.6 | Gm33649 | NC_000085.6 |
| Smyd1 | NC_000072.6 | Rmst | NC_000076.6 |
| Magel2 | NC_000073.6 | Gm38708 | NC_000072.6 |
| Dgkk | NC_000086.7 | Gm13112 | NC_000070.6 |
| Epn3 | NC_000077.6 | Gm38580 | NC_000079.6 |
| Tshz2 | NC_000068.7 | Gm45917 | NC_000077.6 |
| Ctxn3 | NC_000084.6 | Gm13112 | NC_000070.6 |
| Barhl1 | NC_000068.7 | Gm38678 | NC_000075.6 |
| Pax3 | NC_000067.6 | Gm38708 | NC_000072.6 |
| Trpm3 | NC_000085.6 | Lhx1os | NC_000077.6 |
| Pax7 | NC_000070.6 | Gm38678 | NC_000075.6 |
| Samd14 | NC_000077.6 | Gm20554 | NC_000079.6 |
| Pou4f2 | NC_000074.6 | Gm33649 | NC_000085.6 |
| Nkd2 | NC_000079.6 | Gm39507 | NC_000086.7 |
| Lhx1 | NC_000077.6 | Gm38678 | NC_000075.6 |
| Gpr161 | NC_000067.6 | Gm34344 | NC_000068.7 |
| Foxb1 | NC_000075.6 | Gm20554 | NC_000079.6 |
| Igsf1 | NC_000086.7 | Rmst | NC_000076.6 |
| Cdh23 | NC_000076.6 | Gm35102 | NC_000077.6 |
| Shisa6 | NC_000077.6 | Gm36088 | NC_000075.6 |
| Pax3 | NC_000067.6 | Gm33649 | NC_000085.6 |
| Ntf3 | NC_000072.6 | Gm39178 | NC_000074.6 |
| Vipr2 | NC_000078.6 | Gm45917 | NC_000077.6 |
| Gata3 | NC_000068.7 | Lhx1os | NC_000077.6 |
| Ebf3 | NC_000073.6 | Gm35395 | NC_000071.6 |
| Tshz2 | NC_000068.7 | Gm45945 | NC_000077.6 |
| Acot5 | NC_000078.6 | Gm41658 | NC_000084.6 |
| Pax3 | NC_000067.6 | Gm38678 | NC_000075.6 |
| Dgkk | NC_000086.7 | Gm38708 | NC_000072.6 |
| Foxb1 | NC_000075.6 | Gm38708 | NC_000072.6 |
| Shox2 | NC_000069.6 | Gm27151 | NC_000085.6 |
| Tcf7l2 | NC_000085.6 | Gm35395 | NC_000071.6 |
| Fibcd1 | NC_000068.7 | Gm45917 | NC_000077.6 |
| Fibcd1 | NC_000068.7 | Gm29906 | NC_000067.6 |
| Pou4f2 | NC_000074.6 | Gm38678 | NC_000075.6 |
| Irx1 | NC_000079.6 | Gm38678 | NC_000075.6 |
| Epha8 | NC_000070.6 | Gm39507 | NC_000086.7 |
| Epha8 | NC_000070.6 | Gm33649 | NC_000085.6 |
| Dgkk | NC_000086.7 | 4930412O13Rik | NC_000068.7 |
| Cdhr1 | NC_000080.6 | Gm39507 | NC_000086.7 |
| Arhgap36 | NC_000086.7 | Gm33649 | NC_000085.6 |
| Tmem255a | NC_000086.7 | Gm27151 | NC_000085.6 |
| Trpm3 | NC_000085.6 | Gm39507 | NC_000086.7 |
| Epha8 | NC_000070.6 | Gm27151 | NC_000085.6 |
| Medag | NC_000071.6 | C030004G16Rik | NC_000084.6 |
| Adamts19 | NC_000084.6 | Gm44781 | NC_000073.6 |
| Samd14 | NC_000077.6 | Gm38708 | NC_000072.6 |
| Dsc3 | NC_000084.6 | Gm41322 | NC_000081.6 |
| Slc6a12 | NC_000072.6 | 4930412O13Rik | NC_000068.7 |
| Sfrp5 | NC_000085.6 | Gm35395 | NC_000071.6 |
| Tenm3 | NC_000074.6 | Gm42071 | NC_000077.6 |
| Syne4 | NC_000073.6 | Gm39507 | NC_000086.7 |
| Tmem255a | NC_000086.7 | Gm13112 | NC_000070.6 |
| Shox2 | NC_000069.6 | Gm33649 | NC_000085.6 |
| Epn3 | NC_000077.6 | Gm39507 | NC_000086.7 |
| Arhgap36 | NC_000086.7 | Gm27151 | NC_000085.6 |
| Adcy8 | NC_000081.6 | Gm39178 | NC_000074.6 |
| Samd14 | NC_000077.6 | Gm27151 | NC_000085.6 |
| Shox2 | NC_000069.6 | 4930412O13Rik | NC_000068.7 |
| Ctxn3 | NC_000084.6 | Rmst | NC_000076.6 |
| Irx1 | NC_000079.6 | Gm39507 | NC_000086.7 |
| Krt80 | NC_000081.6 | 1700121C08Rik | NC_000070.6 |
| Slc17a6 | NC_000073.6 | Gm38708 | NC_000072.6 |
| Tmem40 | NC_000072.6 | C030004G16Rik | NC_000084.6 |
| Magel2 | NC_000073.6 | Gm38580 | NC_000079.6 |
| Cabp7 | NC_000077.6 | Gm36088 | NC_000075.6 |
| Arhgap36 | NC_000086.7 | 4930412O13Rik | NC_000068.7 |
| Pappa2 | NC_000067.6 | Gm32412 | NC_000070.6 |
| AW551984 | NC_000075.6 | Gm13112 | NC_000070.6 |
| Calb2 | NC_000074.6 | Rmst | NC_000076.6 |
| Cdhr1 | NC_000080.6 | 4930412O13Rik | NC_000068.7 |
| Agt | NC_000074.6 | Rmst | NC_000076.6 |
| Vwc2l | NC_000067.6 | Gm42071 | NC_000077.6 |
| Ctxn3 | NC_000084.6 | Gm20554 | NC_000079.6 |
| Fibcd1 | NC_000068.7 | Gm35102 | NC_000077.6 |
| Dgkk | NC_000086.7 | Gm27151 | NC_000085.6 |
| Calb2 | NC_000074.6 | Gm38678 | NC_000075.6 |
| Dgkk | NC_000086.7 | Gm33649 | NC_000085.6 |
| Prlhr | NC_000085.6 | Gm20554 | NC_000079.6 |
| Ntng1 | NC_000069.6 | Gm39178 | NC_000074.6 |
| Sox14 | NC_000075.6 | Gm44781 | NC_000073.6 |
| Baiap3 | NC_000083.6 | Gm38678 | NC_000075.6 |
| Epha8 | NC_000070.6 | Gm38580 | NC_000079.6 |
| Trpm3 | NC_000085.6 | Rmst | NC_000076.6 |
| Ndst4 | NC_000069.6 | Gm38678 | NC_000075.6 |
| Smyd1 | NC_000072.6 | Gm2516 | NC_000074.6 |
| Adcy8 | NC_000081.6 | Gm29906 | NC_000067.6 |
| Cbln3 | NC_000080.6 | Gm20554 | NC_000079.6 |
| Slc6a12 | NC_000072.6 | Gm2516 | NC_000074.6 |
| Baiap3 | NC_000083.6 | Gm39507 | NC_000086.7 |
| Dgkk | NC_000086.7 | Rmst | NC_000076.6 |
| AW551984 | NC_000075.6 | Gm38708 | NC_000072.6 |
| Foxb1 | NC_000075.6 | Gm35395 | NC_000071.6 |
| Cdh23 | NC_000076.6 | Gm44781 | NC_000073.6 |
| AW551984 | NC_000075.6 | Gm2516 | NC_000074.6 |
| Arhgap36 | NC_000086.7 | Gm2516 | NC_000074.6 |
| Shox2 | NC_000069.6 | Gm13112 | NC_000070.6 |
| Pirt | NC_000077.6 | Gm34344 | NC_000068.7 |
| Irx1 | NC_000079.6 | Gm2516 | NC_000074.6 |
| Shox2 | NC_000069.6 | Gm44781 | NC_000073.6 |
| Samd14 | NC_000077.6 | Gm13112 | NC_000070.6 |
| Epha8 | NC_000070.6 | Gm38678 | NC_000075.6 |
| Tcf7l2 | NC_000085.6 | Gm2516 | NC_000074.6 |
| Prlhr | NC_000085.6 | Gm38708 | NC_000072.6 |
| Cbln3 | NC_000080.6 | 4930412O13Rik | NC_000068.7 |
| Cbln3 | NC_000080.6 | Gm13112 | NC_000070.6 |
| Trpm3 | NC_000085.6 | Gm35395 | NC_000071.6 |
| Lhx9 | NC_000067.6 | Rmst | NC_000076.6 |
| Baiap3 | NC_000083.6 | Gm2516 | NC_000074.6 |
| AW551984 | NC_000075.6 | Gm33649 | NC_000085.6 |
| Tmem255a | NC_000086.7 | Gm38580 | NC_000079.6 |
| Cdhr1 | NC_000080.6 | Gm20554 | NC_000079.6 |
| Gpr161 | NC_000067.6 | Gm45945 | NC_000077.6 |
| Fibcd1 | NC_000068.7 | Gm42071 | NC_000077.6 |
| Pax7 | NC_000070.6 | 4930412O13Rik | NC_000068.7 |
| Lmo1 | NC_000073.6 | Gm33649 | NC_000085.6 |
| Gata3 | NC_000068.7 | Gm35395 | NC_000071.6 |
| Lmo1 | NC_000073.6 | Gm27151 | NC_000085.6 |
| Smyd1 | NC_000072.6 | Gm33649 | NC_000085.6 |
| Nkd2 | NC_000079.6 | Gm33649 | NC_000085.6 |
| AW551984 | NC_000075.6 | Gm27151 | NC_000085.6 |
| Nkd2 | NC_000079.6 | Gm39178 | NC_000074.6 |
| Slc17a6 | NC_000073.6 | Gm38580 | NC_000079.6 |
| Tmem40 | NC_000072.6 | Gm32412 | NC_000070.6 |
| Ctxn3 | NC_000084.6 | 4930412O13Rik | NC_000068.7 |
| Gm29779 | NC_000071.6 | Gm39507 | NC_000086.7 |
| AW551984 | NC_000075.6 | 4930412O13Rik | NC_000068.7 |
| Pax7 | NC_000070.6 | Gm27151 | NC_000085.6 |
| Gata3 | NC_000068.7 | Gm39507 | NC_000086.7 |
| Slc17a6 | NC_000073.6 | Rmst | NC_000076.6 |
| Sfrp5 | NC_000085.6 | Gm39507 | NC_000086.7 |
| Epha8 | NC_000070.6 | 4930412O13Rik | NC_000068.7 |
| Lmo1 | NC_000073.6 | Gm38678 | NC_000075.6 |
| Agt | NC_000074.6 | Gm33649 | NC_000085.6 |
| Arhgap36 | NC_000086.7 | Gm39507 | NC_000086.7 |
| Lhx9 | NC_000067.6 | Gm39507 | NC_000086.7 |
| Tshz2 | NC_000068.7 | Gm29906 | NC_000067.6 |
| Smyd1 | NC_000072.6 | Gm20554 | NC_000079.6 |
| Nkd2 | NC_000079.6 | Gm27151 | NC_000085.6 |
| Agt | NC_000074.6 | Gm2516 | NC_000074.6 |
| Slc6a12 | NC_000072.6 | Gm38580 | NC_000079.6 |
| Cbln3 | NC_000080.6 | Gm38708 | NC_000072.6 |
| Lhx5 | NC_000071.6 | Rmst | NC_000076.6 |
| Nnat | NC_000068.7 | Gm2516 | NC_000074.6 |
| Pirt | NC_000077.6 | Gm34294 | NC_000068.7 |
| Cabp7 | NC_000077.6 | Gm45917 | NC_000077.6 |
| Shisa6 | NC_000077.6 | Gm34344 | NC_000068.7 |
| Lhx1 | NC_000077.6 | Rmst | NC_000076.6 |
| Gpr161 | NC_000067.6 | Gm34294 | NC_000068.7 |
| Vipr2 | NC_000078.6 | Gm36088 | NC_000075.6 |
| Gm29779 | NC_000071.6 | Gm33649 | NC_000085.6 |
| Acot5 | NC_000078.6 | Gm38615 | NC_000071.6 |
| Igsf1 | NC_000086.7 | Gm2516 | NC_000074.6 |
| Prlhr | NC_000085.6 | Gm38580 | NC_000079.6 |
| Sfrp5 | NC_000085.6 | Rmst | NC_000076.6 |
| Vipr2 | NC_000078.6 | Gm34294 | NC_000068.7 |
| Ndst4 | NC_000069.6 | Gm36088 | NC_000075.6 |
| Agt | NC_000074.6 | Gm27151 | NC_000085.6 |
| Tmem255a | NC_000086.7 | Gm38678 | NC_000075.6 |
| Gm29779 | NC_000071.6 | Gm27151 | NC_000085.6 |
| Irx2 | NC_000079.6 | Rmst | NC_000076.6 |
| Pax7 | NC_000070.6 | Gm33649 | NC_000085.6 |
| Cabp7 | NC_000077.6 | Gm34294 | NC_000068.7 |
| Cdhr1 | NC_000080.6 | Gm44781 | NC_000073.6 |
| Epn3 | NC_000077.6 | Rmst | NC_000076.6 |
| Agt | NC_000074.6 | Gm20554 | NC_000079.6 |
| Dgkk | NC_000086.7 | Gm38678 | NC_000075.6 |
| Arhgap36 | NC_000086.7 | Gm38678 | NC_000075.6 |
| Tmem255a | NC_000086.7 | 4930412O13Rik | NC_000068.7 |
| Ctxn3 | NC_000084.6 | Gm38708 | NC_000072.6 |
| Gpr161 | NC_000067.6 | Gm36088 | NC_000075.6 |
| Cdh23 | NC_000076.6 | Gm34294 | NC_000068.7 |
| Pou4f2 | NC_000074.6 | Rmst | NC_000076.6 |
| Bok | NC_000067.6 | Gm40055 | NC_000069.6 |
| Samd14 | NC_000077.6 | Gm38678 | NC_000075.6 |
| Smyd1 | NC_000072.6 | Gm38708 | NC_000072.6 |
| Irx2 | NC_000079.6 | Gm39507 | NC_000086.7 |
| Cdh23 | NC_000076.6 | Gm29906 | NC_000067.6 |
| Calb2 | NC_000074.6 | Gm39507 | NC_000086.7 |
| Prlhr | NC_000085.6 | Gm39507 | NC_000086.7 |
| Plpp4 | NC_000073.6 | Gm39507 | NC_000086.7 |
| Smyd1 | NC_000072.6 | Gm27151 | NC_000085.6 |
| Cabp7 | NC_000077.6 | Gm44781 | NC_000073.6 |
| Ebf3 | NC_000073.6 | Gm39507 | NC_000086.7 |
| Pax3 | NC_000067.6 | Rmst | NC_000076.6 |
| Barhl1 | NC_000068.7 | Rmst | NC_000076.6 |
| Cdhr1 | NC_000080.6 | Rmst | NC_000076.6 |
| Tmem40 | NC_000072.6 | C730002L08Rik | NC_000085.6 |
| Gata3 | NC_000068.7 | Rmst | NC_000076.6 |
| AW551984 | NC_000075.6 | Gm39507 | NC_000086.7 |
| Pappa2 | NC_000067.6 | Gm40055 | NC_000069.6 |
| Lmo1 | NC_000073.6 | 4930412O13Rik | NC_000068.7 |
| Lhx5 | NC_000071.6 | Gm39507 | NC_000086.7 |
| Ebf3 | NC_000073.6 | Rmst | NC_000076.6 |
| Epn3 | NC_000077.6 | Gm35395 | NC_000071.6 |
| Cbln3 | NC_000080.6 | Gm27151 | NC_000085.6 |
| Ndst4 | NC_000069.6 | Gm27151 | NC_000085.6 |
| Fibcd1 | NC_000068.7 | Gm45945 | NC_000077.6 |
| Lmo1 | NC_000073.6 | Gm39507 | NC_000086.7 |
| Bok | NC_000067.6 | Gm32412 | NC_000070.6 |
| Samd14 | NC_000077.6 | 4930412O13Rik | NC_000068.7 |
| Epha8 | NC_000070.6 | Lhx1os | NC_000077.6 |
| Pax7 | NC_000070.6 | Gm44781 | NC_000073.6 |
| Agt | NC_000074.6 | Gm13112 | NC_000070.6 |
| Ctxn3 | NC_000084.6 | Gm44781 | NC_000073.6 |
| Gdpd2 | NC_000086.7 | Gm39178 | NC_000074.6 |
| Syne4 | NC_000073.6 | Gm2516 | NC_000074.6 |
| Nnat | NC_000068.7 | Gm39178 | NC_000074.6 |
| Dgkk | NC_000086.7 | Gm2516 | NC_000074.6 |
| Nkd2 | NC_000079.6 | Gm38678 | NC_000075.6 |
| Prlhr | NC_000085.6 | Lhx1os | NC_000077.6 |
| Epn3 | NC_000077.6 | Gm2516 | NC_000074.6 |
| Cbln3 | NC_000080.6 | Gm33649 | NC_000085.6 |
| Magel2 | NC_000073.6 | Lhx1os | NC_000077.6 |
| Sox14 | NC_000075.6 | Gm27151 | NC_000085.6 |
| Epha8 | NC_000070.6 | Gm2516 | NC_000074.6 |
| Htr4 | NC_000084.6 | Gm34344 | NC_000068.7 |
| Tmem40 | NC_000072.6 | Gm29906 | NC_000067.6 |
| Pappa2 | NC_000067.6 | Gm42071 | NC_000077.6 |
| Tmem40 | NC_000072.6 | Gm35102 | NC_000077.6 |
| 2210418O10Rik | NC_000068.7 | Gm41658 | NC_000084.6 |
| Tmem40 | NC_000072.6 | Gm42071 | NC_000077.6 |
| Tenm3 | NC_000074.6 | Gm40055 | NC_000069.6 |
| Npffr1 | NC_000076.6 | Gm32442 | NC_000078.6 |
| Adamtsl5 | NC_000076.6 | Gm33585 | NC_000077.6 |
| Tenm3 | NC_000074.6 | Gm32442 | NC_000078.6 |
| Pappa2 | NC_000067.6 | Gm35102 | NC_000077.6 |
| Pappa2 | NC_000067.6 | Gm34294 | NC_000068.7 |
| Adcy8 | NC_000081.6 | Gm41658 | NC_000084.6 |
| 2210418O10Rik | NC_000068.7 | C030004G16Rik | NC_000084.6 |
| Adcy8 | NC_000081.6 | Gm40055 | NC_000069.6 |
| Adcy8 | NC_000081.6 | C030004G16Rik | NC_000084.6 |
| Pappa2 | NC_000067.6 | Gm39997 | NC_000068.7 |
| Htr2a | NC_000080.6 | Gm29906 | NC_000067.6 |
| Htr2a | NC_000080.6 | Gm34294 | NC_000068.7 |
| Gm14296 | NC_000068.7 | Gm42071 | NC_000077.6 |
| 2210418O10Rik | NC_000068.7 | Gm40055 | NC_000069.6 |
| Htr2a | NC_000080.6 | Gm35102 | NC_000077.6 |
| Gm14296 | NC_000068.7 | Gm33585 | NC_000077.6 |
