# Supplementary material for: Protective effects and regulatory pathways of melatonin in traumatic brain injury mice model: Transcriptomics and bioinformatics analysis
Source: Front Mol Neurosci. 2022 Sep 9;15:974060. doi: 10.3389/fnmol.2022.974060 (PMC9500234; doi:10.3389/fnmol.2022.974060)
Supplement: Supplementary file 5 [file Table_5.docx]

| Supplementary Table 5. The Degree algorithm results of cytoHubba | |
| --- | --- |
| Degree | |
| Rank | Node |
| 1 | STX1A |
| 2 | SNAP25 |
| 3 | SYT1 |
| 4 | VAMP2 |
| 5 | SLC17A7 |
| 6 | SLC32A1 |
| 7 | SLC18A3 |
| 8 | ATP6V1F |
| 9 | CPLX1 |
| 10 | ATP6V0D2 |
