# Supplementary figures and images for: Protective effects and regulatory pathways of melatonin in traumatic brain injury mice model: Transcriptomics and bioinformatics analysis
Source: Front Mol Neurosci. 2022 Sep 9;15:974060. doi: 10.3389/fnmol.2022.974060 (PMC9500234; doi:10.3389/fnmol.2022.974060)

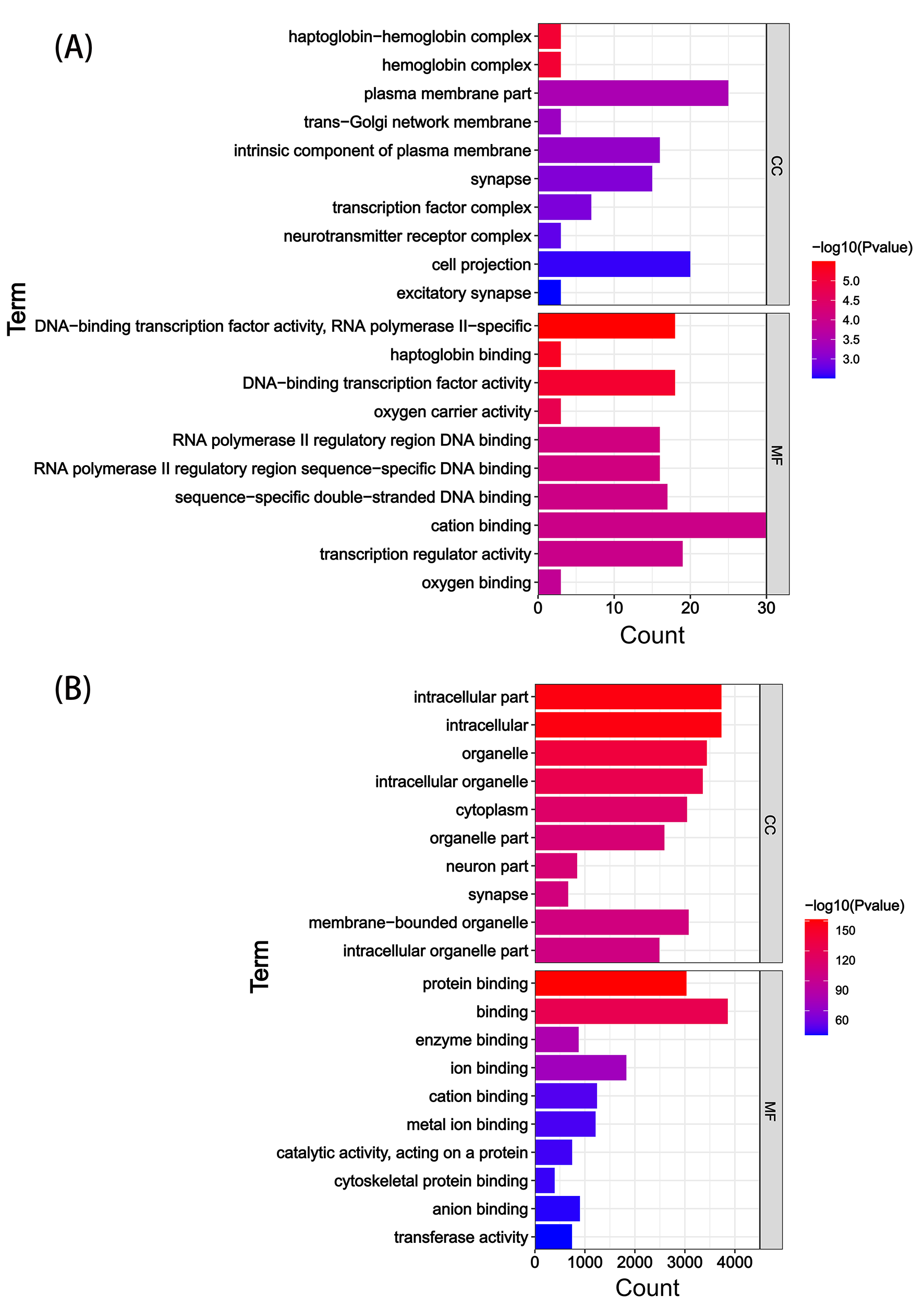

Supplement: Supplementary Figure 1 — GO cellular component and GO_MF analysis results of the DEmRNAs and DElncRNAs. (A) The GO_CC and GO_MF enrichment analysis of the predicted genes of DEmRNAs. The abscissa represents the count in the GO_CC and GO_MF term, the ordinate represents the GO_CC and GO_MF term, and the color of the column represents the -log10 (p-value). (B) The GO_CC and GO_MF enrichment analysis of the predicted genes of DElncRNAs. The abscissa represents the count in the GO_CC and GO_MF term, the ordinate represents the GO_CC and GO_MF term, and the color of the column represents the -log10 (p-value). [file Image_1.TIF]

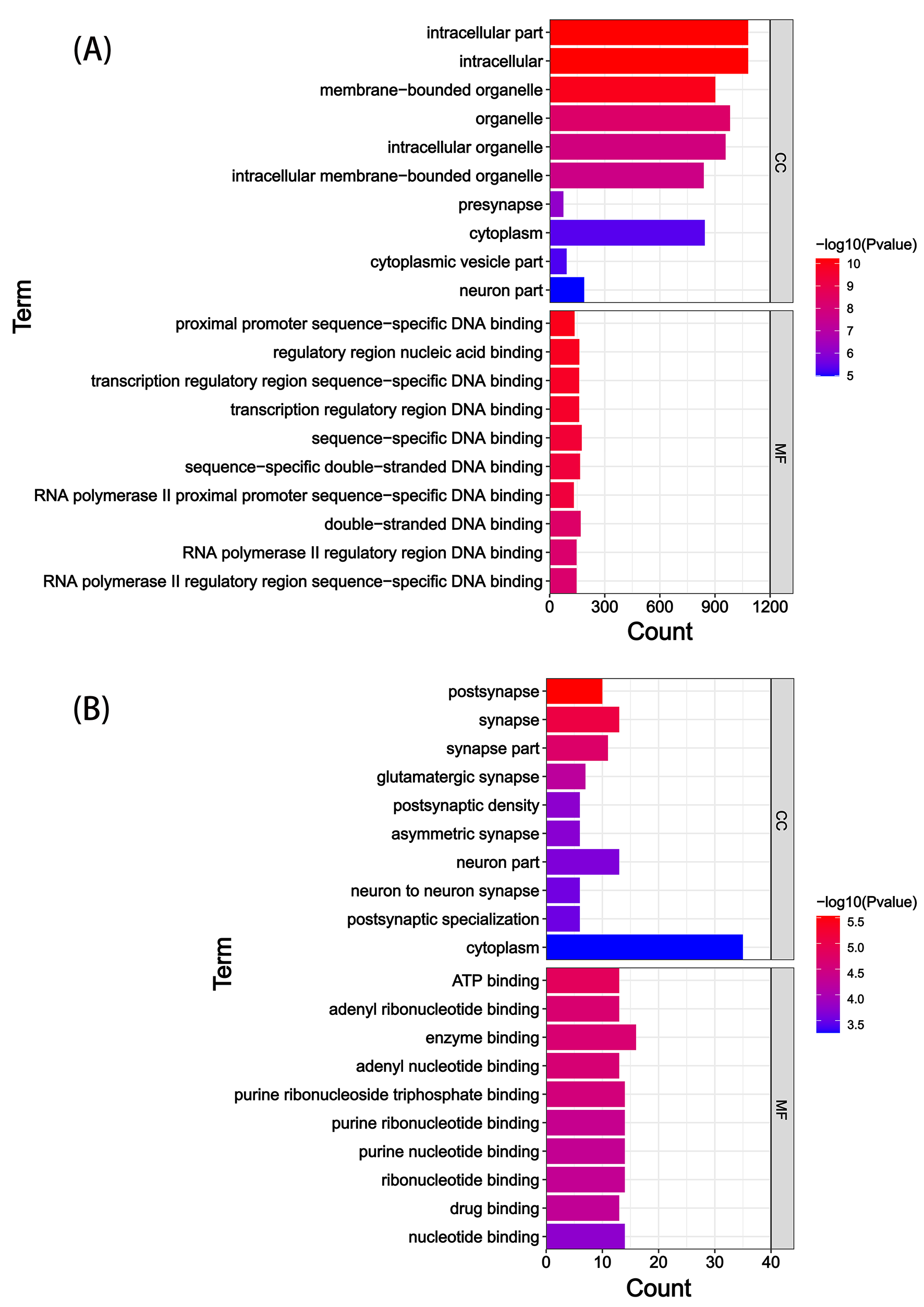

Supplement: Supplementary Figure 2 — GO cellular component and GO_MF analysis results of the DEmiRNAs and DEcircRNAs. (A) The GO_CC and GO_MF enrichment analysis of the predicted genes of DEmiRNAs. The abscissa represents the count in the GO_CC and GO_MF term, the ordinate represents the GO_CC and GO_MF term, and the color of the column represents the -log10 (p-value). (B) The GO_CC and GO_MF enrichment analysis of the predicted genes of DEcircRNAs. The abscissa represents the count in the GO_CC and GO_MF term, the ordinate represents the GO_CC and GO_MF term, and the color of the column represents the -log10 (p-value). [file Image_2.TIF]
